# CD73 expression on effector T cells sustained by TGF-β facilitates tumor resistance to anti-4-1BB/CD137 therapy

**DOI:** 10.1038/s41467-018-08123-8

**Published:** 2019-01-11

**Authors:** Siqi Chen, Jie Fan, Minghui Zhang, Lei Qin, Donye Dominguez, Alan Long, Gaoxiang Wang, Renqiang Ma, Huabin Li, Yi Zhang, Deyu Fang, Jeffrey Sosman, Bin Zhang

**Affiliations:** 1 Department of Medicine-Division of Hematology/Oncology, Robert H. Lurie Comprehensive Cancer Center, Northwestern University Feinberg School of Medicine, Chicago, IL 60611 USA; 2 Biotherapy Center, The First Affiliated Hospital of Zhengzhou University, 450052 Zhengzhou, Henan China; 3 Department of Hematology, Tongji Hospital, Tongji Medical College, Huazhong University of Science and Technology, 430030 Wuhan, China; 4 Department of Allergy Center, Otorhinolaryngology Hospital, The First Affiliated Hospital of Sun Yat-sen University, 510080 Guangzhou, China; 5 Department of Otolaryngology-Head and Neck Surgery, Affiliated Eye, Ear, Nose and Throat Hospital, Fudan University, 200031 Shanghai, China; 6 Department of Pathology, Northwestern University Feinberg School of Medicine, Chicago, IL 60611 USA

## Abstract

Agonist antibodies (Ab) directed against costimulatory molecules on the surface of antigen-primed T cells are in various stages of pre-clinical and clinical trials, albeit with limited therapeutic benefit as single agents. The underlying mechanisms of action remain incompletely understood. Here, we demonstrate an inhibitory role of ecto-enzyme CD73 for agonistic anti-4-1BB/CD137 Ab therapy. In particular, anti-4-1BB treatment preferentially drives CD73^−^ effector T cell response for tumor inhibition. Anti-CD73 neutralizing Ab further improves anti-4-1BB therapy associated with enhanced anti-tumor T cell immunity. However, the TGF-β-rich tumor milieu confers resistance to anti-4-1BB therapy by sustaining CD73 expression primarily on infiltrating CD8^+^ T cells across several tumor models. TGF-β blockade results in downregulation of CD73 expression on infiltrating T cells and sensitizes resistant tumors to agonistic anti-4-1BB therapy. Thus, our findings identify a mechanism of action for more effective clinical targeting of 4-1BB or likely other costimulatory molecules.

## Abstract (web summary)

Targeting the immune-stimulatory receptor 4-1BB has only yielded modest benefit in cancer treatment. In this study, the authors show that CD73 expression on effector T cells sustained by TGF-β drives tumor resistance to anti-4-1BB therapy and therefore TGF- β blockade can be used to overcome such resistance.

## Introduction

Besides blocking immune-inhibitory molecules (e.g., CTLA-4 and PD-1/PD-L1), activating immune co-stimulatory receptors to potentiate antitumor immune responses is a promising approach^1–5^. One such immuno-stimulatory receptor with ongoing clinical applications is 4-1BB (CD137/TNFRSF9). 4-1BB is a member of the tumor necrosis factor receptor (TNFR) superfamily is expressed mainly on activated CD4^+^ and CD8^+^ T cells^6–8^. Although agonist antibodies have been the best studied modality for activating 4-1BB, the capacity of 4-1BB monotherapy to treat advanced tumors is limited. Indeed, targeting 4-1BB with agonist antibodies in the clinic has only yielded modest benefit^3,9,10^. The resistant mechanisms of anti-4-1BB therapy remain to be defined.

Building on the seminal discovery by Sitkovsky et al. which demonstrated tumor protection by adenosine receptor A2AR activation^11^, CD73-mediated adenosinergic effects are now considered one of the important immunosuppressive pathways in the tumor^12–17^. CD73 is a cell surface ecto-enzyme (ecto-5′-nucleotidase) that catalyzes the dephosphorylation of extracellular AMP into adenosine, which in turn activates the G protein–coupled receptors (mainly A2AR and A2BR) to exert potent immunoregulatory activity^18^. CD73 is expressed primarily by the cancer cells and the immune cells such as CD4^+^Foxp3^+^ regulatory T cells (Tregs), and myeloid-derived suppressor cells (MDSCs) that are recruited by the tumor. We and others have demonstrated the pivotal role of tumor and host CD73-mediated adenosinergic effects on tumor growth and metastasis in multiple tumor models^19–23^. Further, a human high-affinity antagonistic antibody, MEDI9447^24^, that non-competitively inhibits CD73 enzymatic activity has been applied in a phase-I clinical trial (NCT02503774).

In this study, we identified a reciprocal regulation of CD73 expression with concomitant CD8^+^ T cell activity by TGF-β and 4-1BB ligation, thereby dictating the efficacy of anti-4-1BB therapy. Our data highlight an important mechanism of action for 4-1BB agonist-mediated cancer immunotherapy.

## Results

### Anti-4-1BB agonist therapy induces tumor regression in CD73^−/−^ mice

**Fig. 1:**
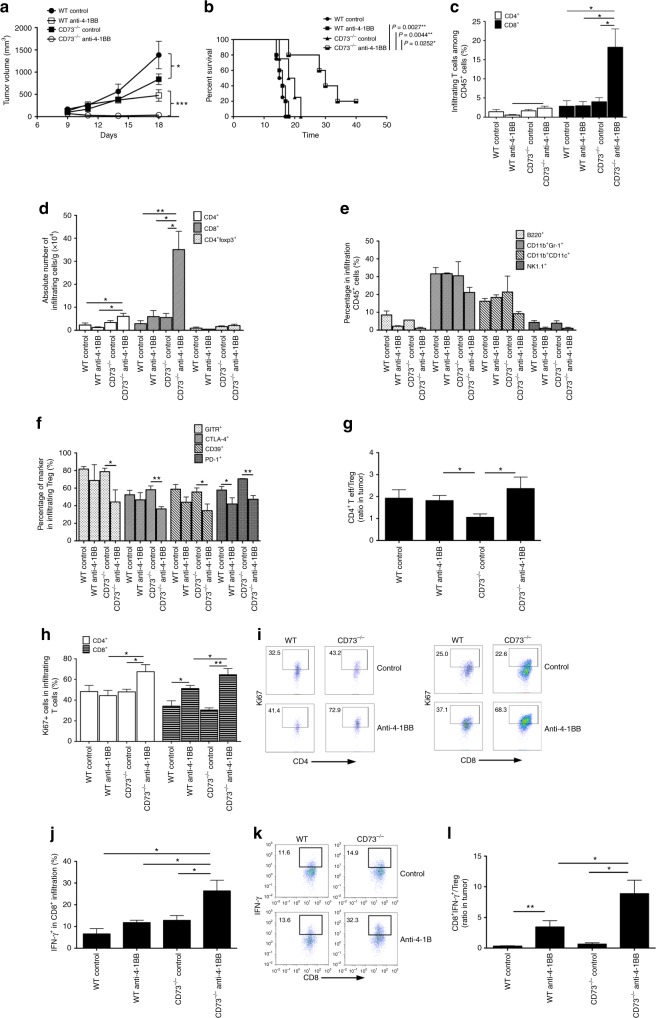
Anti-4-1BB induces tumor regression in CD73 deficient mice. WT and CD73^−/−^ mice were injected s.c. with B16-SIY melanoma cells and treated with anti-4-1BB or control IgG. **a** Tumor size was measured every 2–4 days. **b** Survival curves of B16-SIY-bearing mice (5 mice per group). **c** B16-SIY tumors from treated WT and CD73^−/−^ mice were harvested 18 days after tumor challenge and analyzed by flow cytometry for accumulation of infiltrating CD3^+^TCRβ^+^, CD3^+^CD4^+^ and CD3^+^CD8^+^ T cells. **d** Absolute number of CD4^+^, CD4^+^Foxp3^+^, and CD8^+^ T cells per gram of tumors were also calculated. **e** Percentage of other main immune cell subsets as indicated of total CD45^+^ tumor infiltrates. **f** Percentage of different Treg-associated markers for CD4^+^Foxp3^+^ T cell infiltrates in the treated B16-SIY-bearing WT and CD73^−/−^ mice. **g** Ratio of CD4^+^Foxp3^-^ (effectors) to CD4^+^Foxp3^+^ cells (Tregs) and CD8^+^ to CD4^+^Foxp3^+^ cells. **h** Percentage of Ki67^+^ among tumor-infiltrating CD4^+^ or CD8^+^ T cells and (**i**) representative dot plots. **j** Percentage of IFN-γ^+^ in tumor-infiltrating CD8^+^ T cells, and (**k**) representative dot plots. **l** The ratio of CD8^+^IFN-γ^+^ to CD4^+^Foxp3^+^ Tregs was also calculated. *p < 0.05, **p < 0.01, ***p < 0.001. ANOVA analysis, log-rank test and unpaired Student’s two-tailed *t* test were used. Data (mean ± SEM) are representative of at least 2 independent experiments with 3–5 independently analyzed mice/group

As shown in Fig. 1a, we observed the modest inhibition of tumors in WT hosts with anti-4-1BB treatment similar to that in CD73^−/−^ hosts with control IgG treatment, consistent with the previous results. More importantly, the tumor regression and improved survival were found in the CD73^−/−^ hosts following anti-4-1BB treatment (Fig. 1a, b), suggesting that CD73 expressed by host cells suppresses the antitumor effect of anti–4-1BB therapy in the B16-SIY model. Within tumor microenvironment, CD73^−/−^ hosts with anti-4-1BB treatment recruited the greatest number of T cells especially CD8^+^ T cells compared with other groups (Fig. 1c, d and Supplementary Fig. 1), indicating that B16-reactive CD8^+^ T cells may be accumulating in the tumor. By contrast, anti-4-1BB minimally affected the tumor infiltration of other main immune cell subsets including B cells (B220^+^), myeloid-derived suppressor cells (MDSCs, Gr1^+^CD11b^+^), dendritic cells (DC, CD11b^+^CD11c^+^Gr1^−^), and NK cells (NK1.1^+^) (Fig. 1e). Anti-4-1BB was sufficient to downregulate the expression levels of a number of functional markers on intratumoral Treg cells in CD73^−/−^ hosts, but only one marker (PD1) was changed by anti-4-1BB in WT hosts (Fig. 1f). We further found in CD73^−/−^ hosts, anti-4-1BB significantly increased the ratio of T effector cell (CD4^+^Foxp3^-^) to Treg (CD4^+^Foxp3^+^) cells (Fig. 1g) and induced the higher proliferation of tumor-infiltrating both CD4^+^ and CD8^+^ T cells, as indicated by the expression levels of the cell cycle associated protein Ki67 (Fig. 1h, i). Notably, there was an increased frequency of IFN-γ-secreting CD8^+^ T cells in the tumor in response to anti-4-1BB treatment in CD73^−/−^ hosts (Fig. 1j, k). As a result, the ratio of IFN-γ^+^CD8^+^ cells to Treg was highest in CD73^−/−^ hosts with anti-4-1BB (Fig. 1l). Collectively, these results suggest that host CD73 deficiency in combination with anti-4-1BB therapy enhanced the infiltration of intratumoral effector CD8^+^ T cells while attenuating accumulation of functional Tregs, likely leading to successful regression of B16-SIY tumors.

### Anti-CD73 and anti-4-1BB synergize to mediate tumor regression

**Fig. 2:**
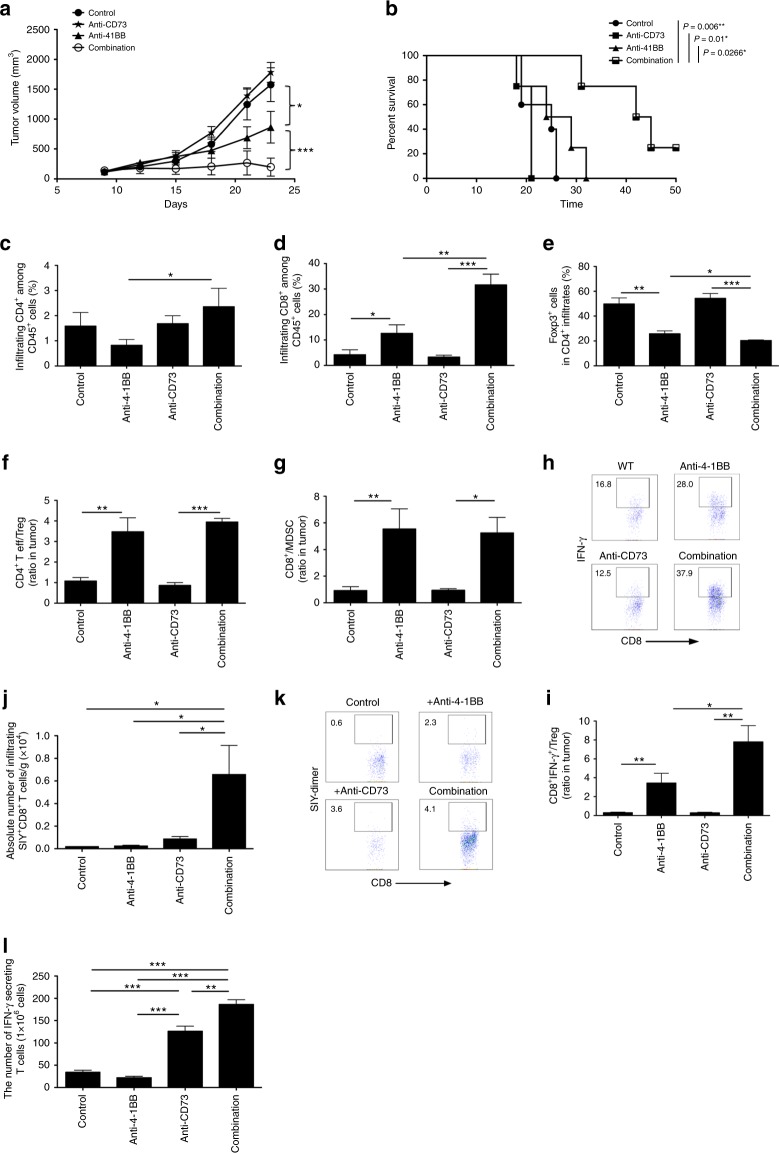
Combination therapy of CD73 blockade and anti-4-1BB facilitates tumor regression. WT mice were injected s.c. with B16-SIY melanoma cells and treated with control IgG, anti-CD73, anti-4-1BB, or both anti-CD73 and anti-4-1BB. **a** Tumor size was measured every 2–3 days (5 mice per group). **b** Survival curves of B16-SIY-bearing mice in another independent experiment treated as indicated (8 mice per group). Percentage of CD4^+^ (**c**) or CD8^+^ (**d**) among tumor-infiltrating CD45^+^ T cells, and percentage of Foxp3^+^ (**e**) among total tumor-infiltrating CD4^+^ T cells in the treated B16-SIY-bearing mice as indicated. Ratios of CD4^+^Foxp3^-^ (effectors) to CD4^+^Foxp3^+^ (Tregs) cells (**f**), and CD8^+^ to Gr1^+^CD11b^+^ MDSCs (**g**). Representative flow dot plots for percentage of IFN-γ^+^ in tumor-infiltrating CD8^+^ T cells (**h**), and the ratio of CD8^+^IFN-γ^+^ to CD4^+^Foxp3^+^ Tregs (**i**). **j** Detection of infiltrating SIY-specific CD8^+^ T cells by dimer staining from B16-SIY-bearing mice treated as indicated and (**k**) representative flow dot plots. **l** ELISPOT analysis of IFN-γ secreting CD8^+^ T cells from DLN of B16-SIY-bearers treated as indicated in the presence of SIY peptides (3 mice per group). The total number was counted. *p < 0.05, **p < 0.01, ***p < 0.001. ANOVA analysis, log-rank test and unpaired Student’s two-tailed t test were used. Data (mean ± SEM) are representative of at least 2 independent experiments

In a clinically relevant setting, mice receiving anti-CD73 treatment alone failed to exhibit the significant anti-tumor advantage over the control mice, but anti-4-1BB monotherapy delayed tumor growth (Fig. 2a). Strikingly, compared to each therapy alone, combination therapy resulted in a superior inhibition of tumor growth, and induced tumor regression in 2 of 5 mice at least at day 23 (Fig. 2a). Combination therapy led to a great increase in survival compared with other groups (Fig. 2b). To explore the cellular mechanisms mediating tumor inhibition in the anti-4-1BB/anti-CD73 system, we analyzed the immune cell distribution within the B16-SIY tumors by flow cytometry. As shown in Fig. 2c, d, combination therapy resulted in an increase in the percentage of tumor-infiltrating CD4^+^ T cells, compared to anti-4-1BB. The percentage of CD8^+^ T cells among whole CD45^+^ tumor infiltrates was significantly higher in mice treated with combination therapy than that in mice with each therapy alone. Conversely, we observed a significant decrease in the percentage of tumor infiltrating Foxp3^+^ Treg cells among CD4^+^ population in anti-4-1BB/anti-CD73-treated mice, compared with anti-4-1BB-treated mice alone (Fig. 2e). In spite of a slight increase in the intratumoral MDSC infiltration, the percentage of CD11b^+^CD11c^+^ DCs remained unchanged in the anti-4-1BB/CD73-treated mice (not depicted). Ratios of intratumoral CD4^+^Foxp3^−^T effector cells to Treg (Fig. 2f), and CD8^+^ T cells to MDSCs (Fig. 2g) were markedly elevated with anti-4-1BB treatment. To determine if infiltrating CD8^+^ T cells were functional, we analyzed their intracellular expression of IFN-γ. The increase in CD8^+^IFN-γ^+^ T cell infiltration (Fig. 2h) was further reflected in the dramatic increase in the ratio of CD8^+^IFN-γ^+^ T cells to Treg cells (Fig. 2i) at the tumor site by anti-4-1BB in comparison with combination therapy, which correlated with improved tumor inhibition by combination therapy (Fig. 2a, b). Similarly, we also observed that anti–4-1BB/anti-CD73 combination therapy increased effector CD8^+^IFN-γ^+^ T cell accumulation in spleen of tumor-bearing mice (Supplementary Fig. 2). To examine further tumor antigen-specific T cell response, we found a significant increase in the number of tumor SIY-specific CD8^+^ T cells from combination therapy compared with control groups by dimer staining (Fig. 2j, k). There were also a greater number of SIY-induced IFN-γ-producing CD8^+^ T cells in the DLN cells from combination therapy than control groups (Fig. 2l). Importantly, to exclude the intrinsic effect of the tumor size on treatment modality, we analyzed the immune cell distribution within the B16-SIY tumors at similar sizes early following treatment. As expected, the percentage of tumor-infiltrating CD8^+^ T cells among whole CD45^+^ population was significantly elevated in mice treated with combination therapy as compared with mice with monotherapy (Supplementary Fig. 3A). Likewise, the number of SIY-induced IFN-γ-producing CD8^+^ cells from DLN (Supplementary Fig. 3B) and infiltrating CD8^+^ IFN-γ^+^ T cell (Supplementary Fig. 3C) were significantly increased in mice treated with combination therapy compared with control groups. In addition, we also confirmed synergistic antitumor effect of anti–GITR/anti-CD73 combination therapy, which was supported by a significant increase in antitumor T cell immunity (Supplementary Fig. 4). These data suggest that combination of CD73 blockade and targeting of 4-1BB or other costimulatory molecules augments the number of effector CD8^+^ T cells, leading to a qualitatively more effective anti-tumor T cell response against the growth of established tumors.

### Anti-4-1BB drives preferential expansion of CD73^−^CD8^+^ T cell subset

**Fig. 3:**
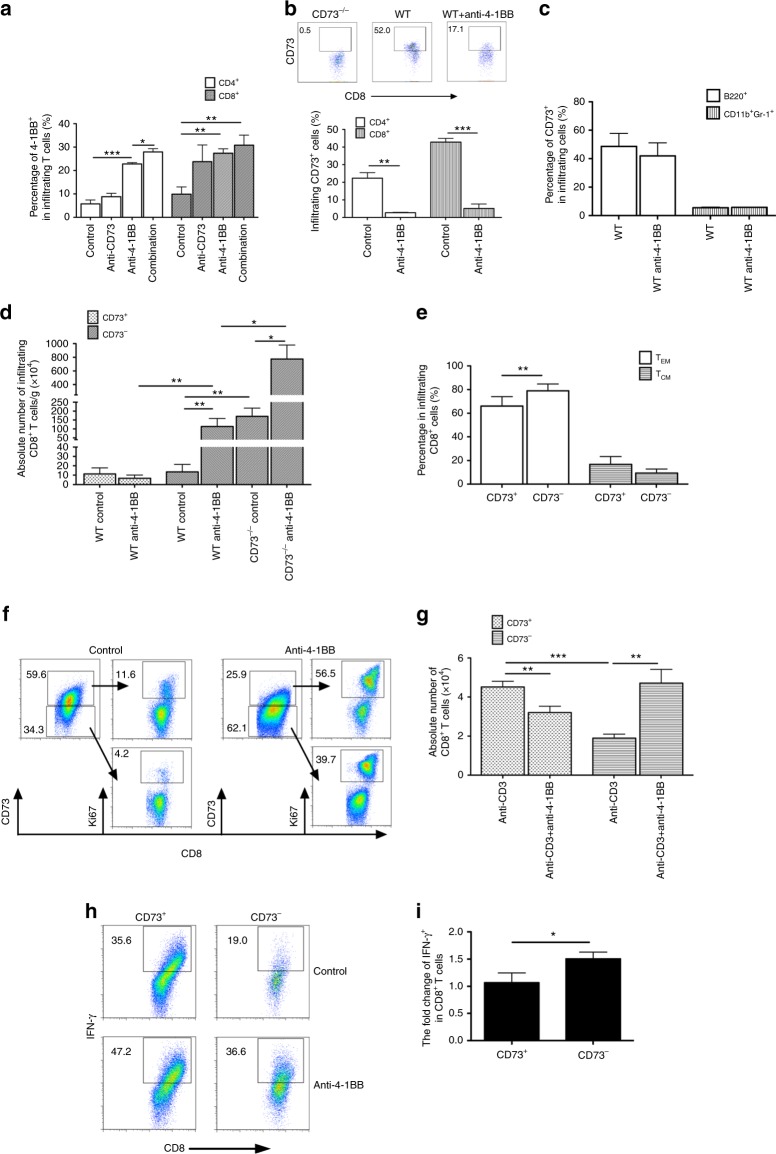
Anti-4-1BB preferentially drives the expansion of CD73-negative CD8^+^ T cell subset. Percentage of 4-1BB^+^ cells (**a**), or CD73^+^ cells (**b**) among infiltrating CD4^+^ and CD8^+^ T cells in B16-SIY tumor-bearing mice treated as indicated was determined by flow cytometry (5 mice per group). **c** Percentage of CD73^+^ cells among tumor-infiltrating B220^+^ cells or Gr1^+^CD11b^+^ MDSCs in B16-SIY-bearing mice treated as indicated. **d** The absolute number of CD73^+^ versus CD73^−^ subsets from tumor-infiltrating CD8^+^ T cells were calculated in B16-SIY-bearing mice treated with control IgG or anti-4-1BB. **e** Percentage of T_EM_ (CD62L^-^CD44^+^), or T_CM_ (CD62L^+^CD44^+^) in CD73^+^ versus CD73^-^ subsets from B16-SIY-infiltrating CD8^+^ T cells treated with anti-4-1BB. Purified CD8^+^ T cells from naïve mice were treated with control IgG or anti-4-1BB in the presence of anti-CD3/anti-CD28 in vitro. The expression levels of CD73 or Ki67 in CD8^+^ T cells were evaluated by flow cytometry. **f** The representative dot plots are depicted. **g** The absolute number of CD73^+^ versus CD73^-^ subsets from cultured CD8^+^ T cells was also counted. **h** The representative dot plots showed the percentage of IFN-γ^+^ cells in CD73^+^ versus CD73^-^ subsets from CD8^+^ T cells. **i** The fold changes of IFN-γ^+^ cells in CD73^+^ versus CD73^-^ subsets were calculated. *p < 0.05, **p < 0.01, ***p < 0.001. Unpaired and paired Student’s two-tailed *t* test were used. Data (mean ± SEM) are representative of 2 independent experiments with 5 independently analyzed mice/group

We examined the interaction between the CD73 expression and 4-1BB-mediated costimulation in the tumor-bearing hosts 18 days after tumor cell injection. Anti-4-1BB treatment significantly increased the frequency of 4-1BB^+^ cells among both infiltrating CD4^+^ and CD8^+^ T cells and combination treatment further increased the 4-1BB^+^ cells in infiltrating CD4^+^ but not in CD8^+^ T cells (Fig. 3a). Interestingly, anti-4-1BB treatment reduced the percentage of CD73^+^ cells among both infiltrating CD4^+^ and CD8^+^ T cells (Fig. 3b) but not other types of immune cells tested (e.g., B cells and MDSCs) (Fig. 3c) in B16-SIY-bearing mice. There was no significant difference in the number of CD73^+^ versus CD73^−^ subsets among tumor-infiltrating CD8^+^ T cells from WT mice receiving control IgG. However, anti-4-1BB exclusively increased the accumulation of CD73^−^negative subset in WT mice (Fig. 3d), which was also confirmed in B16-SIY tumors at similar sizes 14 days following treatment (Supplementary Fig. 3D). Notably, the largest number of intratumoral CD8^+^ T cells were observed in the CD73^−/−^ hosts with anti-4-1BB treatment (Fig. 3d). Furthermore, there were more effector memory CD8^+^ T cells in CD73^−^subset after anti-4-1BB treatment compared with CD73^+^ subset within tumors (Fig. 3e). Given the known effect of 4-1BB costimulation on CD8^+^ T cell expansion^7^, we examined if anti-4-1BB drives preferentially the proliferation of CD73^−^CD8^+^ T cells in an in vitro setting. There were more CD73^+^ cells than CD73^−^ cells in the CD8^+^ T cells after TCR-mediated (anti-CD3/anti-CD28) stimulation, in line with the enhanced proliferating capacity (Ki67^+^) of CD73^+^ subset compared to the CD73^−^ subset (Fig. 3f, g). With the further 4-1BB-mediated costimulation, however, there was a 10-fold increase in the percentage of Ki67^+^ in CD73^-^ subset in contrast to a 5^-^fold increase in the percentage of Ki67^+^ in CD73^+^ subset. In addition, we found a significant reduction in the number of CD73^+^ subset but a concomitant increase in CD73^−^ subset in response to the 4-1BB costimulation (Fig. 3f, g), which strongly supported our findings in vivo (Fig. 3d). As expected, 4-1BB costimulation resulted in more production of IFN-γ from CD73^−^ subset than CD73^+^ subset (Fig. 3h, i). Thus, our data reveal that downregulation of CD73 expression on T cells by anti-4-1BB is largely attributed to a preferential expansion of CD73-negative T cell compartment with the enhanced IFN-γ production.

### CD73 expression on T cells limits 4-1BB-mediated anti-tumor activity

**Fig. 4:**
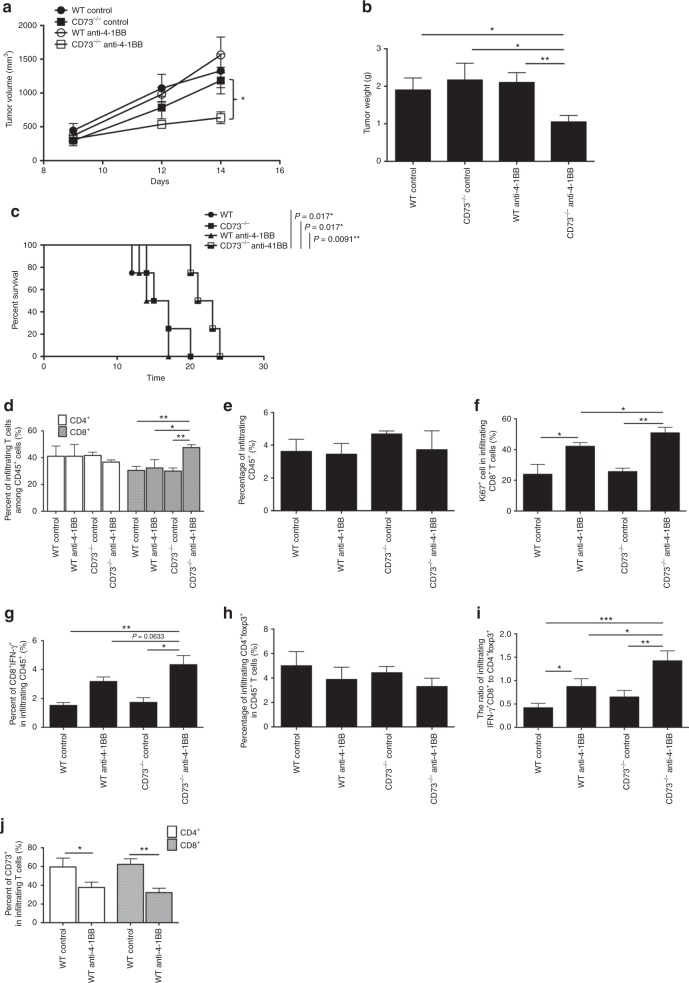
CD73 deficiency on T cells is sufficient to improve anti-4-1BB therapy. B16-SIY tumor-bearing Rag1^−/−^ mice receiving WT or CD73^−/−^ pan-T cells were treated with control IgG or anti-4-1BB. **a** Tumor size was measured every 2–3 days. **b** Tumor weight was measured 14 days after tumor challenge. **c** Survival curves of these B16-SIY-bearing mice. **d** Percentage of CD4^+^ or CD8^+^ T cells among total CD45^+^ tumor infiltrates. **e** Percentage of CD45^+^ cells among total tumor infiltrates. **f** Percentage of Ki67^+^ in tumor-infiltrating CD8^+^ T cells and percentage of CD73^+^ in tumor-infiltrating CD4^+^ or CD8^+^ T cells were also determined. Percentage of CD8^+^IFN-γ^+^ (**g**) or CD4^+^Foxp3^+^ Tregs (**h**) among total CD45^+^ tumor infiltrates. **i** The ratio of CD8^+^IFN-γ^+^ to CD4^+^Foxp3^+^ Tregs was calculated in treated B16-SIY-bearing Rag1^−/−^ mice. **j** Percentage of CD73^+^ in tumor-infiltrating CD4^+^ or CD8^+^ T cells in treated B16-SIY-bearing Rag1^−/−^ mice. *p < 0.05, **p < 0.01. ANOVA analysis, log-rank test and unpaired Student’s two-tailed *t* test were used. Data (mean ± SEM) are representative of 2 independent experiments with 3–5 independently analyzed mice/group

We next asked if CD73 expression on T cells is sufficient to inhibit the 4-1BB-induced antitumor effect. As expected, the hosts receiving both CD73^−/−^ T cells and anti-4-1BB developed the smallest tumors (Fig. 4a, b) and longest survival (Fig. 4c) among all treated groups. Consistently, we found a significant increase in the accumulation of intratumoral CD8^+^ T cells in mice receiving both CD73^−/−^ T cells and anti-4-1BB treatment compared with other three groups (Fig. 4d) while all mice had similar percentages of CD45^+^ tumor infiltrates (Fig. 4e), which was supported by our further observation that these CD8^+^ T cells from this group gained the highest levels of proliferating capacity (Ki67^+^) (Fig. 4f). The ratio of CD8^+^ IFN-γ^+^ to Tregs was dramatically elevated by the anti-4-1BB treatment in the tumor (Fig. 4g–i) and spleen (Supplementary Fig. 5) from CD73^−/−^ group compared to other groups. In line with the aforementioned results (Fig. 3b), anti-4-1BB indeed resulted in a significant decrease in the percentage of CD73^+^ cell in both transferred CD4^+^ and CD8^+^ T cells in the tumor (Fig. 4j) and spleen (Supplementary Fig. 5) from tumor-bearing Rag1^−/−^ hosts, indicating again a specific potent downregulation of CD73 expression on T cells by the 4-1BB-mediated costimulation.

### CD73 ablation impairs the Treg expansion in response to anti-4-1BB

Due to a significant decrease in the percentage of infiltrating Foxp3^+^ Treg cells (Fig. 2e) among CD4^+^ population in mice treated with anti-4-1BB/anti-CD73 compared with mice with anti-4-1BB alone, we tested the role of CD73 in the regulation of Treg accumulation in the face of anti-4-1BB. Adoptive transfer of WT or CD73^−/−^ CD4^+^CD25^−^ naïve T cells (CD45.2^+^) into MC38-bearing mice (CD45.1^+^) was performed for measurement of the in vivo Treg conversion. We found that anti-4-1BB failed to affect the de novo Treg (CD45.2^+^) induction in mice receiving WT versus CD73^−/−^ CD4^+^ T cells. However, anti-4-1BB increased the percentage of recipient Tregs (CD45.1^+^) was in both groups (Supplementary Fig. 6A). Similar data were obtained in the setting of adoptive transfer of CD73^+/−^ OT-II or CD73^−/−^ OT-II CD4^+^CD25^-^ naïve T cells (CD90.2^+^) into B16-OVA-bearing mice (CD90.1^+^) for examining the tumor antigen-specific Treg conversion (Supplementary Fig. 6B), consistent with the previous results^25–27^. We subsequently found that anti-4-1BB inhibited CD73^−/−^ Treg induction but promoted WT Treg induction (Supplementary Fig. 6C). Furthermore, anti-4-1BB decreased the expression levels (MFI) of Foxp3 in CD73^−/−^ CD4^+^ T cells, but not in the WT CD4^+^ T cells (Supplementary Fig. 6C). Similarly, anti-4-1BB increased the proliferation (eflour670 dilution) of WT Tregs but decreased that of CD73^−/−^ Tregs (Supplementary Fig. 6D), which may explain the least accumulation of Treg in the cultures (Supplementary Fig. 6C). Likewise, blockade of A2AR activity by ZM241365 diminished the accumulation and expansion of Tregs induced by anti-4-1BB (Supplementary Fig. 6E). Thus, these data suggest the role of CD73 on Tregs in maintaining the Foxp3 expression and the expansion induced by anti-4-1BB.

### TGF-β-rich tumors resistant to anti-4-1BB therapy

**Fig. 5:**
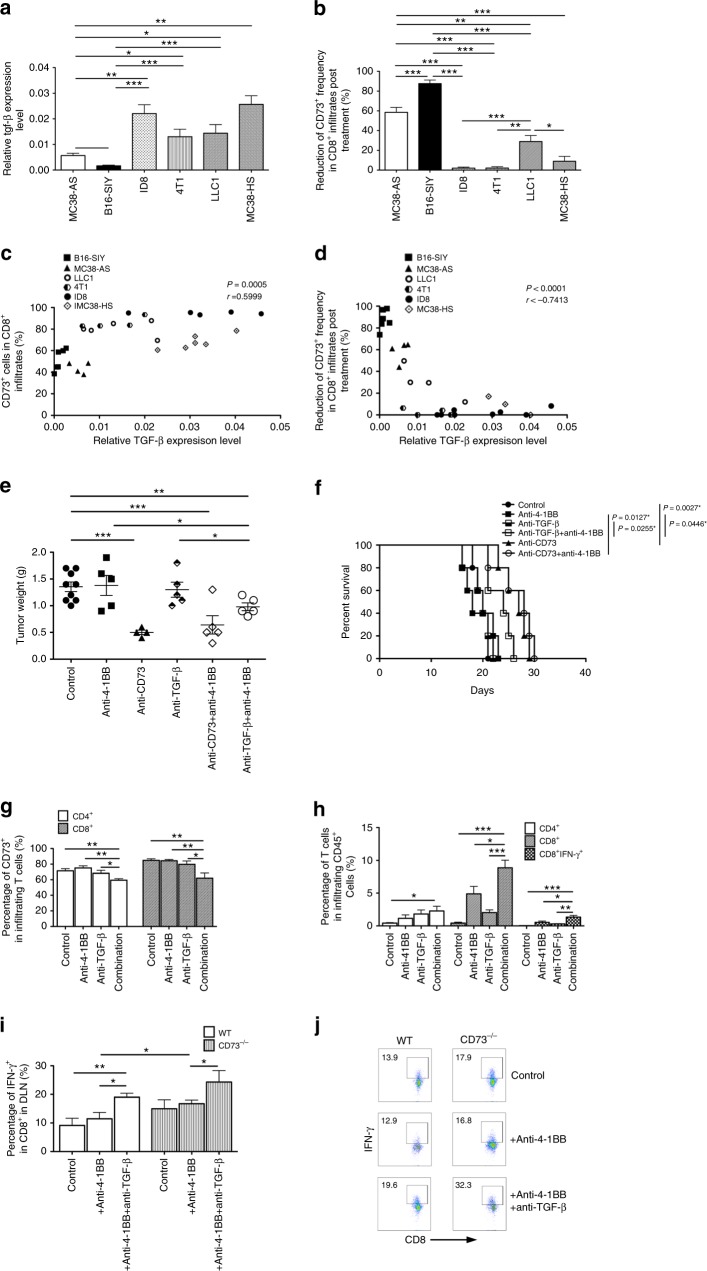
TGF-β-rich tumors sustain CD73 experssion on T cells to hinder anti-4-1BB therapy. **a** The expression levels of TGF-β were analyzed from single cell suspension of different tumor tissues by real-time RT-PCR. **b** The ability of anti-4-1BB treatment to reduce CD73^+^ subset in CD8^+^ tumor infiltrates was calculated across a variety of tumors. **c** The frequency of CD73^+^ subset in CD8^+^ tumor infiltrates was positively correlated with intratumoral expression levels of TGF-β. **d** The ability of anti-4-1BB treatment to reduce CD73^+^ subset in CD8^+^ tumor infiltrates was negatively correlated with intratumoral expression levels of TGF-β. **e** 4T1-bearing mice were treated as indicated. The tumor weight was measured 20 days after tumor challenge. **f** Survival curves of 4T1-bearing mice (5 mice per group). **g** Percentage of CD73^+^ in tumor-infiltrating CD4^+^ or CD8^+^ T cells in treated 4T1-bearing mice as indicated. **h** Percentage of CD4^+^, CD8^+^, or CD8^+^IFN-γ^+^ T cells among total CD45^+^ tumor infiltrates in treated 4T1-bearing mice as indicated. **i** Detection of IFN-γ^+^ cells among transferred CD8^+^ T cells in MC38-HS-bearing mice treated, and (**j**) representative flow dot plots. *p < 0.05, **p < 0.01, ***p < 0.001. Log-rank test and unpaired Student’s two-tailed *t* test were used. Data (mean ± SEM) are representative of 2 independent experiments with 5 independently analyzed mice/group

Given the essence of TGF-β in maintaining CD73 expression on leukocytes^28,29^, we asked if TGF-β could sustain CD73 expression on effector CD8^+^ T cells, thereby keeping them resistant to anti-4-1BB. To test this, we found the low levels of TGF-β were observed specifically in the B16-SIY and MC38-AS models in contrast to the marked elevated levels of TGF-β in the other tumor models (ID8, 4T1, LLC1, and MC38-HS) (Fig. 5a), which is in line with the ability of anti-4-1BB treatment to reduce CD73^+^ subset in CD8^+^ tumor infiltrates (Fig. 5b). Notably, the frequency of CD73^+^ subset in CD8^+^ tumor infiltrates was positively correlated with intratumoral expression levels of TGF-β (Fig. 5c) that was further negatively correlated with the ability of anti-4-1BB treatment to reduce CD73^+^ subset in CD8^+^ tumor infiltrates (Fig. 5d). These data suggest a potential involvement of TGF-β in the resistant mechanism of anti-4-1BB therapy through induction of CD73 expression. To confirm this hypothesis, in the TGF-β-rich 4T1 tumor model we found that TGF-β blockade using neutralizing anti-TGF-β mAb restored the 4-1BB-elicited potent antitumor activity as comparable to anti-CD73 monotherapy (Fig. 5e, f), whereas neither anti-TGF-β or anti-4-1BB alone was able to inhibit tumor growth (Fig. 5e, f). Consistent with these, combination of anti-TGF-β/anti-4-1BB but not each therapy alone decreased the frequency of CD73^+^ subset among both intratumoral CD4^+^ and CD8^+^ T cells (Fig. 5g). Furthermore, we observed a dramatic increase in the percentage of 4T1 tumor-infiltrating CD8^+^ T cells especially CD8^+^IFN-γ^+^ T cells in the anti-4-1BB/TGF-β-treated mice compared with the other groups (Fig. 5h). Similar results were also found in additional TGF-β-rich models of LLC1 (Supplementary Fig. 7A, B), ID8 (Supplementary Fig. 7C, D), and MC38-HS (Supplementary Fig. 7E, F), in which anti-4-1BB treatment was insufficient (LLC1) or unable (ID8 and MC38-HS) to alter the frequency of CD73^+^ subset in tumor-infiltrating CD8^+^ T cells, and combination of anti-CD73/anti-4-1BB failed to induce the synergistic/additive anti-tumor effect. In contrast, in the TGF-β-low MC38-AS tumor model, similar to the B16-SIY model we found a synergistic antitumor effect of combination of anti-CD73/anti-4-1BB therapy (Supplementary Fig. 8A) accompanied by the enhanced accumulation of intratumoral CD8^+^ T cells producing IFN-γ (Supplementary Fig. 8B, C). As expected, anti-4-1BB resulted in a significant reduction in the frequency of CD73^+^ subset in CD8^+^ T cell infiltrates (Supplementary Fig. 8D) but a concomitant increase in the frequency of CD73^−^subset preferentially producing IFN-γ (Supplementary Fig. 8E, F). Moreover, we found no synergy between TGF-β blockade and anti-4-1BB for the control of B16-SIY tumor growth (Supplementary Fig. 8G) and survival (Supplementary Fig. 8H). To test the importance of CD73 expression on T cells for TGF-β-mediated inhibition to anti-4-1BB, WT or CD73^−/−^ CD8^+^ T cells (CD45.2^+^) were adoptively transferred into CD45.1^+^ hosts bearing the TGF-β-rich MC38-HS tumors. We found that CD73^−/−^ CD8^+^ T cells produced more IFN-γ than WT CD8^+^ T cells after anti-4-1BB treatment. Furthermore, anti-TGF-β treatment increased the levels of IFN-γ from either transferred WT CD8^+^ T cells or CD73^−/−^ CD8^+^ T cells in response to anti-4-1BB treatment (Fig. 5i, j). These data indicate that the 4-1BB-elicited anti-tumor effect is likely dependent on relative reduction of CD73^+^ effector T cells. However, tumors with high expression levels of TGF-β sustain CD73 expression on T cells, leading to the tumor resistance to anti-4-1BB therapy.

### TGF-β impedes the 4-1BB-mediated CD8^+^ T cell activity

**Fig. 6:**
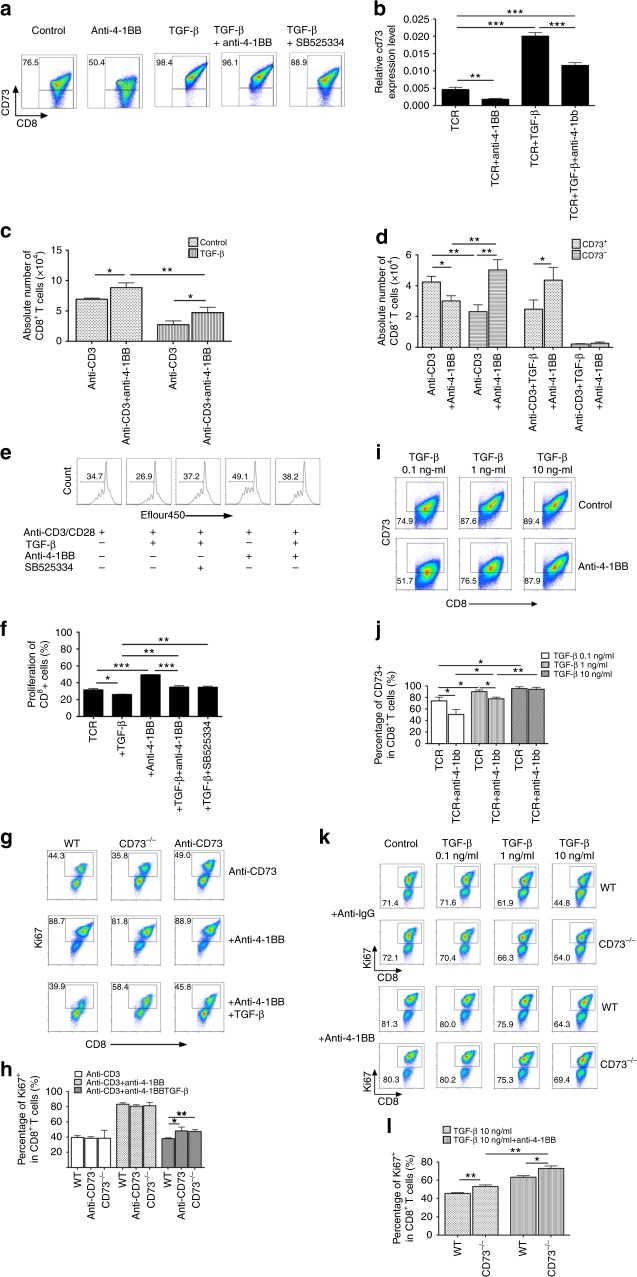
TGF-β sustains CD73 expression and mitigates 4-1BB-mediated expansion of CD8^+^ T cells. **a** Purified CD8^+^ T cells were cultured as indicated in the presence of anti-CD3/anti-CD28. After 3 d, the percentage of CD73^+^ in CD8^+^ T cells was measured by flow cytometer. **b** The mRNA expression of CD73 was measured by real-time RT-PCR. Splenocytes were cultured with indicated reagents in the presence of anti-CD3. After 3 days, the absolute number of total CD8^+^ T cells (**c**), CD73^+^ versus CD73^-^ subsets (**d**) among these treated CD8^+^ T cells were counted. Purified CD8^+^ T cells labeled with efluor450 were cultured in different doses of 5 ng/ml TGF-β with control IgG or anti-4-1BB in the presence of anti-CD3/anti-CD28. The representative flow histograms (**e**) and summarized results (**f**) of T cell proliferation indicated by efluor450 dilution were analyzed. **g**, **h** Flow analysis of percentage of Ki67^+^ in WT or CD73^−/−^ CD8^+^ T cells cultured with indicated reagents in the presence of anti-CD3 with/without 5 ng/ml TGF-β. Flow analysis of percentage of CD73^+^ (**i**, **j**), or Ki67^+^ (**k**, **l**) in WT or CD73^−/−^ CD8^+^ T cells cultured with indicated reagents in the presence of anti-CD3/anti-CD28 with/without different doses of TGF-β (0.1–10 ng/ml). *p < 0.05, **p < 0.01, ***p < 0.001. Unpaired Student’s two-tailed *t* test was used. Data (mean ± SEM) are representative of 2 independent experiments

As expected, anti-4-1BB alone decreased CD73 expression in CD8^+^ T cells at both protein (Fig. 6a) and gene (Fig. 6b) expression levels. However, this 4-1BB-mediated effect was significantly abrogated by addition of TGF-β (Fig. 6a, b). In line with these, TGF-β inhibited the expansion in the absolute number of CD8^+^ T cells induced by anti-4-1BB treatment (Fig. 6c). Interestingly, we analyzed the number of CD73^+^ versus CD73^−^ subsets among CD8^+^ T cells. After TGF-β treatment, majority of CD8^+^ T cells converted to the CD73^+^ compartment (Fig. 6d). Anti-4-1BB induced the accumulation of CD8^+^CD73^−^ subset only in the absence of TGF-β, but failed in the presence of TGF-β. On the contrary, anti-4-1BB reduced the accumulation of CD8^+^CD73^+^ subset in the absence of TGF-β, but increased in the presence of TGF-β (Fig. 6d). We next verified the importance of TGF-β-mediated signaling in this process. Notably, in the presence of TGF-β, addition of a selective inhibitor of TGF-βR1 SB525334 efficiently recovered the proliferation (indicated by dilution of eflour450) to the basal level (Fig. 6e, f). Similar to the murine cell system, we confirmed the ability of TGF-β to sustain CD73 expression and further inhibit the 4-1BB-induced expansion of human CD8^+^ T cells (Supplementary Fig. 9). We next asked if CD73 ablation could antagonize the inhibitory effect of TGF-β specifically in the presence of anti-4-1BB. Indeed, CD8^+^ T cells with CD73 ablation (genetic deficiency or anti-CD73 mAb) achieved higher expression levels of Ki67 compared with CD73^−^competent CD8^+^ T cells in the presence of both anti-4-1BB and TGF-β (Fig. 6g, h). To test if the inhibitory effect of TGF-β on the 4-1BB-mediated activity is dose-dependent, we cultured purified CD8^+^ T cells in the different concentrations of TGF-β. At a low dose of TGF-β (0.1 ng/ml), anti-4-1BB retained the ability to downregulate CD73 on CD8^+^ T cells; the 4-1BB-mediated effect became weaker when the dose of TGF-β was increased to 1 ng/ml; and a higher dose of TGF-β (10 ng/ml) made these T cells fully resistant to anti-4-1BB (Fig. 6i, j). These results strongly support the notion that TGF-β-rich tumors override the ability of anti-4-1BB to downregulation of CD73 on intratumoral effector T cells (Fig. 5d). In addition, TGF-β inhibited proliferation of both WT and CD73^−/−^ CD8^+^ T cells in a dose-dependent manner, but this effect was diminished at least partially by anti-4-1BB treatment (Fig. 6k). CD73^−/−^ CD8^+^ T cells showed enhanced T cell proliferation capacity by TCR stimulation with anti-4-1BB compared to WT CD8^+^ T cells even in the presence of high-dose TGF-β (Fig. 6l). We also confirmed these results using gp100-specific Pmel CD8^+^ T cells (Supplementary Fig. 10A, B).

Either anti-GITR or anti-OX40 treatment reduced the CD73^+^ subset among the CD8^+^ T cells (Supplementary Fig. 11A), and this was also reflected by a preferential expansion of CD73^−^ subset versus CD73^+^ subset (Supplementary Fig. 11B). Furthermore, we observed that high levels of TGF-β counteracted the GITR or OX40 costimulation by sustaining expression of CD73 on these T cells in a dose-dependent fashion (Supplementary Fig. 11C). The results indicate that interactions between costimulation of TNFR family molecules and TGF-β-mediated CD73 expression on T cells are not confined only to 4-1BB.

### TGF-β-STAT3 axis overrides 4-1BB-mediated effect on CD8^+^ T cells

**Fig. 7:**
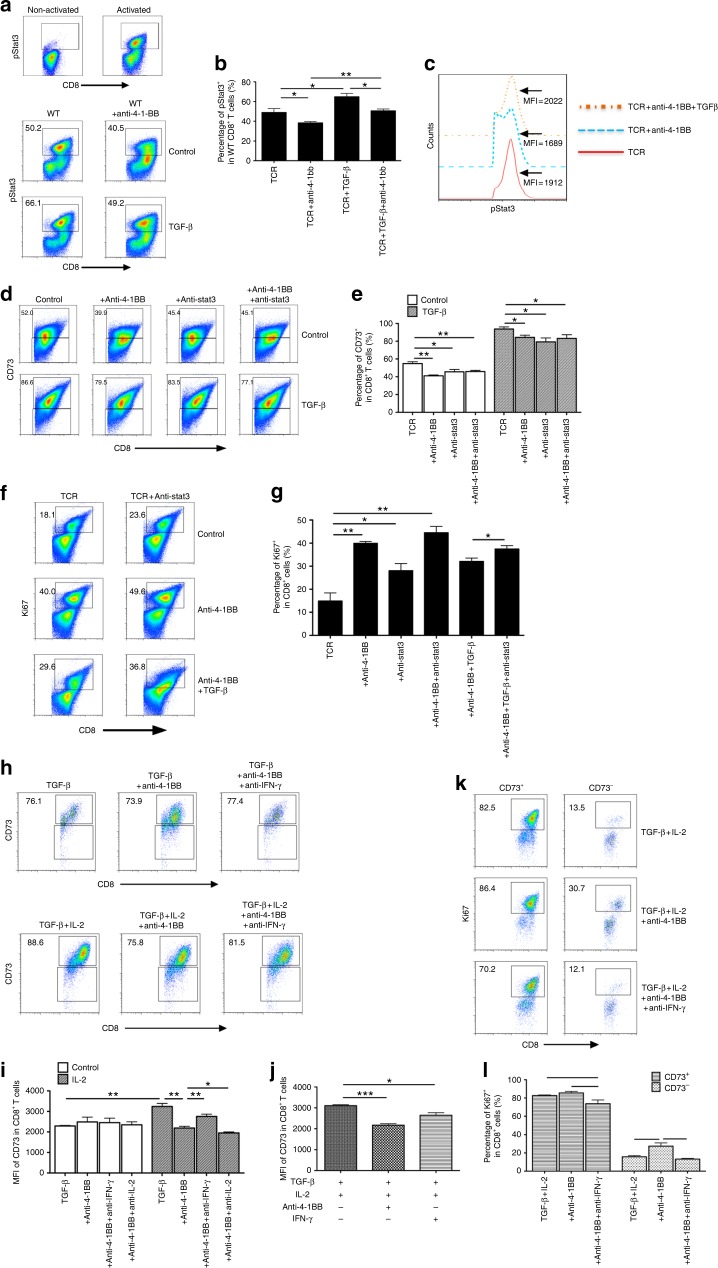
The reciprocal regulation of CD73 and expansion of CD8^+^ T cells by TGF-β and 4-1BB ligation. **a**, **b** Purified CD8^+^ T cells were cultured with TGF-β, anti-4-1BB, or control IgG in the presence of anti-CD3/anti-CD28. After 3 days, intracellular expression of phosphorylated STAT3 (pSTAT3) on CD8^+^ T cells was measured by flow cytometry. **c** The MFI of pSTAT3 in CD8^+^ T cells was determined by flow cytometry. CD8^+^ T cells were treated with indicated reagents as in **a**, in the presence or absence of WP1066 (STAT3 inhibitor). The percentage of CD73^+^ (**d**, **e**) or Ki67^+^ (**f**, **g**) in CD8^+^ T cells was measured by flow cytometry. **h** T cells were cultured with indicated reagents in the presence of anti-CD3 for 3 days. CD73 expression on CD8^+^ T cells was analyzed by flow cytometry. **i**, **j** The MFI of CD73 expression on CD8^+^ T cells under indicated conditions as in **h**. **k**, **l** Percentage of Ki67^+^ in CD8^+^CD73^+^ versus CD8^+^CD73- subsets under indicated conditions as in **h** was compared by flow cytometry. *p < 0.05, **p < 0.01, ***p < 0.001. Unpaired Student’s two-tailed *t* test was used. Data (mean ± SEM) are representative of 2 independent experiments

As STAT3 is a critical transcription factor that drives CD73 expression^29^, we tested the role of STAT3 in the control of CD73 expression on CD8^+^ T cells in the interaction between TGF-β and 4-1BB ligation. We found that anti-4-1BB resulted in a significant decrease in both percentage (Fig. 7a, b) and MFI (Fig. 7c) of phosphorylated STAT3 (pSTAT3) expression compared with the control IgG. However, this 4-1BB-elicited effect was attenuated by addition of TGF-β (Fig. 7a, b). Similar to anti-4-1BB, STAT3 blockade using a selective inhibitor, WP1066 mediated CD73 downregulation; but failed to further affect CD73 expression in the presence of anti-4-1BB. To a lesser extent, the effect was also observed in the presence of TGF-β (5 ng/ml) that increased the overall basal levels of CD73 (Fig. 7d, e). These data suggest that pSTAT3 is likely a convergent downstream target of TGF-β and 4-1BB-mediated reciprocal regulation of CD73 on T cells. Consistent with the results on CD73 expression regulation, either anti-4-1BB treatment or STAT3 inhibition alone augmented the Ki67 expression in T cells, while STAT3 inhibition could not further promote 4-1BB-mediated Ki67 expression in T cells (Fig. 7f, g). Although in the presence of TGF-β, STAT3 inhibition further augmented 4-1BB-mediated Ki67 expression in T cells, the Ki67 expression levels in these T cells were still lower than those in T cells treated with STAT3 inhibitors + anti-4-1BB without TGF-β (Fig. 7f, g), suggesting that pSTAT3 is involved at least partly in the TGF-β and 4-1BB-mediated reciprocal regulation of T cell proliferation.

### Reciprocal regulation of CD73 on CD8^+^ T cells by TGF-β and 4-1BB ligation

It has been reported that both IL-2 and IFN-γ are involved in the 4-1BB-mediated T cell activity^30–32^. In the absence of IL-2, TGF-β-sustained CD73 expression was not influenced by addition of anti-4-1BB plus anti-IFN-γ or anti-IL-2 (Fig. 7h, i). Although IL-2 further increased the CD73 expression in the presence of TGF-β, this effect was diminished by anti-4-1BB treatment (Fig. 7h, i). Blockade of IFN-γ but not IL-2 using specific neutralizing mAb further abrogated the anti-4-1BB-mediated downregulation of CD73 expression in the presence of IL-2 and TGF-β (Fig. 7h, i). Moreover, addition of IFN-γ without 4-1BB costimulation was sufficient to reduce CD73 expression in the presence of TGF-β and IL-2, similar to the effect of anti-4-1BB treatment (Fig. 7j), suggesting the importance of 4-1BB-mediated IFN-γ production in the control of CD73 expression on T cells. We also examined the proliferating capacity of CD73^−^ versus CD73^+^ subsets among CD8^+^ T cells in the settings above. Consistently, anti-4-1BB drove the proliferation of CD73^−^ but not CD73^+^ subsets in the presence of TGF-β and IL-2 (Fig. 7k, l). And this 4-1BB-induced effect was abrogated completely by anti-IFN-γ (Fig. 7k, l). We also confirmed these results using gp100-specific Pmel CD8^+^ T cells (Supplementary Fig. 10C-E). Our data indicate the role of both IL-2 and IFN-γ in protecting the 4-1BB-mediated CD73 downregulation and concomitant expansion of effector T cells from TGF-β.

## Discussion

We demonstrate that CD73 expression on T cells sustained by TGF-β in the tumor microenvironment is responsible for mediating resistance to the therapeutic activity of agonist antibodies against a TNFR superfamily member across several tumor types. Our study also highlights a mechanism of action in the TNFR family for agonist therapy and suggests that the reciprocal interaction of TGF-β-induced CD73 with 4-1BB costimulatory signals in T cells may be a promising target for both positively and negatively manipulating the anti-tumor immune response (summarized in Supplementary Fig. 12).

Anti–4-1BB therapy unveiled promising but limited clinical benefits in early clinical trials. Our preclinical results show that CD73 is highly involved in the resistance mechanism of anti-4-1BB treatment. We found that administration of anti-4-1BB antibody combined with the blockade of CD73 achieved the regression of established melanoma tumors. Tumor regression by the anti-4-1BB/anti-CD73 combination therapy was associated with enhanced infiltration of competent IFN-γ^+^CD8^+^ T cells while reduced Treg accumulation, which led to a shift in the balance of effector T cells to Treg cells in the tumors. Neutralization and adoptive transfer studies revealed that superior antitumor activity by combination therapy was dependent on the presence of the T cells, and CD73 expression on T cells directly impaired the 4-1BB-elicited anti-tumor effect. Although the mechanism by which anti-4-1BB treatment regulates Treg has been somewhat controversial^25–27,33–35^, we found that 4-1BB co-stimulation increased committed Treg expansion but decreased in the absence/blockade of CD73. Given the importance of CD73 for Treg activity, anti-CD73 and 4-1BB costimulation may advantageously inhibit functional Treg accumulation to facilitate anti-tumor T cell immunity. In addition, anti-4-1BB treatment did not affect the Foxp3^+^ Treg conversion from Foxp3^−^CD4^+^ T cells in tumor-bearing mice regardless of CD73 expression. Further studies will thus be needed to investigate the fate of CD73^+^ Treg cells and its relevance to anti-tumor activity in anti-4-1BB–treated tumor-bearing hosts.

The protumorigenic function of CD73 on tumor cells and immunosuppressive cells such as Tregs^20,21^ and MDSCs^36–38^ has been well documented. In anti-4-1BB–treated B16-SIY melanoma-bearing mice, the frequency of CD73^+^ cells among both tumor-infiltrating CD4^+^ and CD8^+^ T cells was considerably reduced, which was explained largely by a favorable expansion of CD73^−^ T cell compartment in response to 4-1BB ligation. Indeed, 4-1BB costimulation directly drove preferential proliferation of CD73^−^ subset over CD73^+^ subset among CD8^+^ T cells in vitro. Moreover, 4-1BB costimulation induced higher levels of IFN-γ production from these CD73^−^CD8^+^ T cells, consistent with the inhibitory effect of CD73 on effector T cell activity. As downregulation of CD73 expression levels was observed exclusively in T cell compartment (e.g., not in B cells or myeloid cells) by anti-4-1BB treatment, it implies that 4-1BB agonist could function as a tumor suppressor by preferentially inducing CD73^−^CD8^+^ effector T cell responses against malignancies. It is notable that 4-1BB costimulation can still stimulate the proliferation of CD73^+^ subset, however, to a much lesser extent. The lower levels of 4-1BBL expression on CD73^+^ subset than CD73^+^ subset (not depicted) are the most likely explanations of the differential ability of these 2 subsets to respond to 4-1BB ligation.

Unlike B16-SIY and MC38-AS models, we found that anti-4-1BB slightly reduced the frequency of CD73^+^ T cell infiltrates in the LLC1 lung cancer model, but failed to make alteration in MC38-HS, ID8 ovarian and 4T1 breast tumor models. Combination of CD73 blockade and anti-4-1BB treatment in these tumor models cannot achieve synergistic/additive antitumor effect. Interestingly, compared with B16-SIY and MC38-AS tumors, LLC1, MC38-HS, ID8 and 4T1 tumors expressed significantly higher expression levels of TGF-β, which is known to induce and sustain CD73 expression on leukocytes^28,29^. These results are compatible with a model in which the TGF-β-rich tumor milieu is sufficient to maintain CD73 expression on T cells in mediating tumor resistance to anti-4-1BB therapy. TGF-β-mediated CD73 expression on “regulatory” CD8^+^ effector T cells limits their ability to eliminate tumors, similar to the immunosuppressive mechanism described for CD73 on Tregs and MDSCs. This is supported by the fact that TGF-β blockade restored the downregulation of CD73 expression on intratumoral T cells induced by anti-4-1BB treatment in both 4T1 and MC38-HS tumor models. Furthermore, neither anti-TGF-β or anti-4-1BB therapy exerted significant anti-tumor activity, but mice showed considerable response to combination therapy. Consistent with these data, we showed that TGF-β inhibited the 4-1BB-elicited proliferation of CD8^+^ T cell with sustained CD73 expression in a dose-dependent manner in vitro. As expected, TGF-β blockade rendered CD8^+^CD73^−^ subset to favorably respond to 4-1BB costimulation, and CD73-deficient CD8^+^ T cells retained susceptibility to 4-1BB agonists even in the presence of TGF-β. These findings together provide strong evidence to support a TGF-β-rich tumor milieu in mediating resistance to anti-4-1BB therapy through a mechanism that is at least partially dependent on CD73 on effector T cells.

Previous work has demonstrated that TGF-β along with STAT3 activation induces expression of CD73 on Th17 cells^29^. In this regard, we found both CD73 expression and STAT3 phosphorylation were downregulated in CD8^+^ T cells by anti-4-1BB treatment but were upregulated by addition of TGF-β in vitro. Moreover, inhibition of STAT3 activation diminished the ability of TGF-β to maintain CD73 expression, leading to concomitant improvement of 4-1BB-triggered CD8^+^ T cell expansion. Thus, it is possible that the induction of CD73 expression constitutes a common immunoregulatory mechanism mediated by STAT3-activating cytokines in T cells to counteract 4-1BB costimulation. On the other hand, it has been reported that TGF-β responses could be reversed by 4-1BB costimulation during in vitro differentiation of naive CD8^+^ T cells into effector CTL cells^39^. Indeed, we also showed that 4-1BB costimulation drove CD8^+^ T cell proliferation with corresponding downregulation of CD73 expression and STAT3 phosphorylation in the presence of low levels of TGF-β, but the increased concentrations of TGF-β abrogated the 4-1BB-mediated effect. Taken together, our results then raise the intriguing notion that the reciprocal regulation of tumor TGF-β and 4-1BB costimulation may determine the efficacy of anti-4-1BB therapy in part through STAT3-mediated CD73 expression on effector T cells. Further studies in this area will be important to assess whether other signaling pathway (e.g., HIF-1α, is also known to induce CD73^38,40^) does lead to any functional response involving interaction with 4-1BB ligation to boost anti-tumor T cell activity.

While our findings suggest that 4-1BB-mediated CD73 downregulation in favor of effector T cell response is regulated via the TGF-β-STAT3 pathway, our study also showed the importance of IL-2 and 4-1BB-induced IFN-γ in this process, as demonstrated by our in vitro settings with addition of recombinant IFN-γ/IL-2 and neutralization of these cytokines using blocking antibodies. Therefore, TGF-β-dependent fine-tuning of 4-1BB signaling contributes to “regulatory” CD73^+^CD8^+^ T cell responses via two different aspects: (i) the signal intensity of the STAT3 pathway and (ii) the levels of IFN-γ and IL-2 production in the tumor. These two mechanisms may act in concert to modulate the 4-1BB-elicited anti-tumor effect in vivo.

The results here are of great interest when considering the activity of other TNFR superfamily members in addition to 4-1BB. To this end, we found that anti-CD73/anti-GITR combination therapy induced B16-SIY tumor regression. Moreover, either anti-GITR or anti-OX40 treatment preferentially stimulated the expansion of CD73- compartment among CD8^+^ T cells in vitro, and the effect was further attenuated by addition of TGF-β in a dose-dependent manner. Our data raise the likelihood that interactions between costimulation of TNFR family molecules and TGF-β-mediated CD73 expression on T cells are not confined only to 4-1BB. This is also supported by a preclinical study reporting that the small molecule TGF-β signaling inhibitor synergizes with anti-OX40 to elicit a potent anti-tumor effect against established tumors^41,42^, although it did not elucidate the underlying mechanism. It still remains to be established whether tumor suppressor CD73 is implicated as a common resistant mechanism to immunostimulatory agonistic cancer therapy.

## Methods

### Mice, cell lines, and reagents

C57BL/6 Rag1^−/−^, Pmel, and CD90.1 mice were purchased from the Jackson Laboratory, OT-II Rag1^−/−^ mice were purchased from Taconic. CD73^−/−^ mice were generated by Dr. Linda Thompson^43^. Dr. Hans Schreiber (University of Chicago) provided the 4T1, B16F10, MC38-HS and B16-SIY cell lines. B16F10 cells were infected with MIGR1-ovalbumin (OVA)-IRES-eGFP^19^, and OVA-expressing cells (B16-OVA) were sorted based on GFP expression. OVA production was confirmed by ELISA. An aggressive ID8 line (ID8agg) by serial passage through WT mice^44^ was provided by Dr. Tyler Curiel (University of Texas Health Science Center at San Antonio). MC38-AS cell line was obtained from Dr. Arlene Sharpe (Harvard Medical School). All the cell lines were routinely tested for mycoplasma infections by culture and DNA stain and maintained in complete medium composed of RPMI 1640 with 5% FBS. All animal experiments were approved by institutional animal use committees of Northwestern University. Phospho-STAT3 mAb was purchased from Cell Signaling Technology. All other mAbs for flow cytometry and cytokines were purchased from eBioscience and BioLegend. Proliferation dye eFluor 450 and eFluor 670 were from eBioscience. Depleting mAb clone TY/23 (anti-CD73, BE0209), clone 1D11.16.8 (anti-TGF-β, BE0057) and agonist mAb clone 3H3 (anti-4-1BB, BE0239) were purchased from BioXCell. ZM241385 (A2AR antagonist), SB525334 (TGF-β inhibitor) and WP1066 (STAT3 inhibitor) were from Tocris Bioscience.

### Analysis of cells by flow cytometry

All samples were initially incubated with 2.4G2 to block antibody binding to Fc receptors. Single-cell suspensions were stained with 1 μg of relevant mAbs and then washed twice with cold PBS. Foxp3 staining was performed according to the manufacturer’s instructions (eBioscience). For SIY-specific dimer staning, 2Kb-lg Dimer X was loaded with 40 M excess of the SIY peptide and cells were stained according to the manufacturer’s instruction (BD Bioscience). Intracellular IFN-γ staining under stimulation with 50 ng/ml PMA, 5 μg/ml ionomycin plus 10 μg/ml BFA in the presense of the relevant tumor antigen peptides, was performed according to the manufacturer’s instruction (BD Bioscience)^20^. For pSTAT3 staining, cells were pretreated with IL-6 for 10 mins and then 10^6^ cells were stained with surface markers and fixed in 4% formaldehyde for 10 min at room temperature. Cells were washed with ice-cold PBS containing 2% BSA, followed by the second wash step with ice-cold PBS. Cells were resuspended in 80% methanol and incubated for 30 min at −20 °C. The pellet was washed twice with ice-cold PBS. Anti-pSTAT3 was added in a final volume of 100 ml of ice-cold PBS and incubated at 4 °C for 45 min. Cells were washed with ice-cold PBS containing 2% BSA and analyzed by flow cytometry. Samples were conducted on a MACS-Quant Analyzer (Miltenyi Biotec) and LSRII, and data were analyzed with FlowJo software.

### ELISPOT assay

For detection of IFN-γ-secreting CD8^+^ T cells, DLN cells from B16-SIY-bearing mice were harvested day 14 or day 18 after tumor cell injection and stimulated with/without SIY peptides ex vivo with 2 × 10^5^ cells/well. 48 h later, an ELISPOT assay was performed according to the manufacturer’s instruction (eBioscience)^45^. The numbers and diameters of spots were counted in triplicates and calculated by an automatic ELISPOT counter.

### RNA extraction and real-time PCR

Total RNA was extracted using Trizol reagent (Invitrogen) according to the manufacturer’s instructions. The cDNA synthesis was performed using SuperScript One-Step RT-PCR (Invitrogen). Quantitative real-time PCR was used to quantify genes by SYBR Green (Bio-Rad), and relative abundance of each mRNA was normalized to GAPDH mRNA. The primers used for q-RT-PCR are: *CD73* F: 5’-CTGGGGCACTCTGGTTTTGA-3’;

R: 5′-TCCCCGCAGGCACTTCTTTG-3′;

*TGF-β* F: 5′-CGGGTCTACTATGCTAAAGAGGTCAC-3′;

R: 5′-TTTCTCATAGATGGCGTTGTTGC-3′.

### Treg induction in vivo and in vitro

Splenic CD73^+/+^ OT-II or CD73^−/−^ OT-II CD4^+^CD62L^+^ naive T cells were selected with IMag CD4 magnetic particles (BD Biosciences) and injected i.v. at 5 × 10^6^ per mouse into CD90.1 mice followed by a s.c. injection of 10^6^ B16-OVA cells. The conversion of transferred T cells to Foxp3^+^ cells (CD90.2^+^) in DLN and spleen from B16-OVA tumor–bearing mice was measured by flow cytometer 14 days after tumor cell injection. For the setting using the non-transgenic T cells, splenic CD73^+/+^ or CD73^−/−^ CD4^+^CD62L^+^ naive T cells were injected i.v. at 5 × 10^6^ per mouse into CD45.1 mice followed by a s.c. injection of 10^6^ MC38-HS cells. For the in vitro Treg induction, splenocytes from WT and CD73^−/−^ mice were cultured with 1 μg/ml anti-CD3, 10 ng/ml IL-2, and 5 ng/ml TGF-β for 5 days in the absence or presence of 20 μg/mL anti-4-1BB, and induced CD25^+^Foxp3^+^ cells among total CD4^+^ T cells were subsequently determined by flow cytometry.

### In vitro cell treatment

For the in vitro treatment of CD8^+^ T cells purified from WT, CD73^−/−^, or Pmel mice, anti-CD3/CD28 (1 μg/ml), anti-4-1BB (20 μg/ml) and/or TGF-β (5 ng/ml or as indicated) were added. After three days, cells were counted and analyzed for T cell proliferation (Ki67 expression or eFluor450/670 dilution), CD73 expression and STAT3 phosphorylation by flow cytometry.

### Tumor challenge and treatments

B16-SIY, B16-OVA, MC38-AS, MC38-HS, ID8 or 4T1 cells (10^6^) in suspension were injected s.c. into mice. For the mAb therapy, 7 or 9 days after tumor cell injection, mice were injected i.p. with anti-CD73 (100 μg/mouse), anti-TGF-β (150 μg/mouse), anti-4-1BB (100 μg/mose) or control anti-IgG (100 μg/mouse) once every three days. To examine the role of CD73 on T cells, splenic CD90.2^+^ Pan-T cells from WT or CD73^−/−^ mice were selected with a CD90.2 isolation kit (StemCell Technologies) and injected i.v. at 1 × 10^7^ per mouse into Rag1^−/−^ mice followed by a s.c. injection of 10^6^ B16-SIY cells. Cell transfer and administration of anti-4-1BB or control anti-IgG were repeated every three days. Tumor volumes were measured along 3 orthogonal axes (a, b, and c) and calculated as abc/2 every 2–4 days. Two weeks later, the percentages and phenotype of transferred T cells in spleen, DLN and tumor microenvironment were determined by flow cytometry.

### Statistical analysis

Mean values were compared using an unpaired or paired Student’s two-tailed *t* test. The statistical differences in tumor growth between groups of mice were determined by ANOVA analysis. The statistical differences between the survival of groups of mice were calculated according to the log-rank test. Probability values > 0.05 were considered non-significant.

## Supplementary information

Supplementary Information

## Data Availability

All data are available from the authors upon reasonable request.
