# Supplementary material for: CD73 expression on effector T cells sustained by TGF-β facilitates tumor resistance to anti-4-1BB/CD137 therapy
Source: Nat Commun. 2019 Jan 11;10:150. doi: 10.1038/s41467-018-08123-8 (PMC6329764; doi:10.1038/s41467-018-08123-8)
Supplement: Supplementary file 1 — Supplementary Information [file 41467_2018_8123_MOESM1_ESM.pdf]

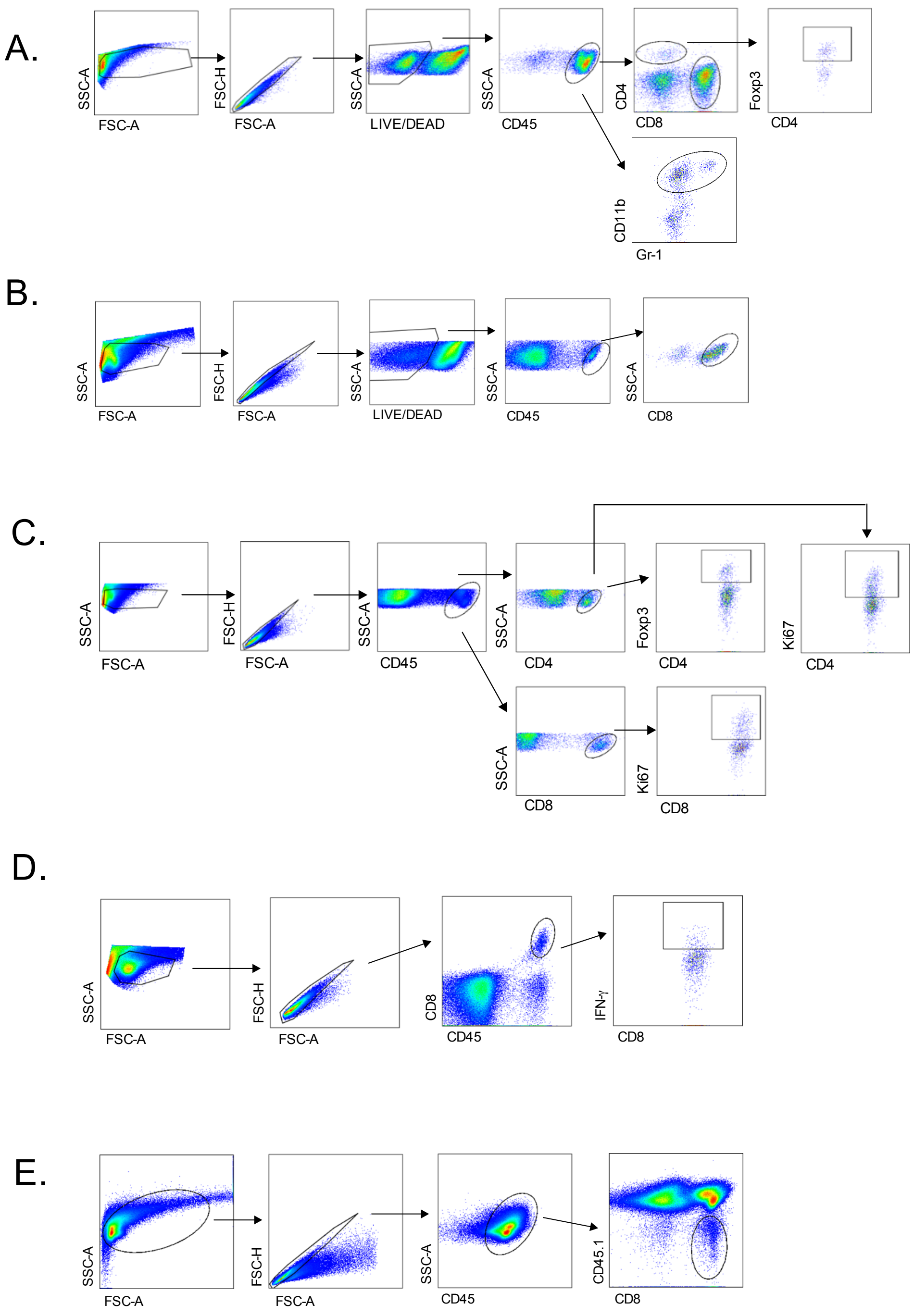

Supplementary Figure 1. FACS sequential gating strategies. (A) Gating strategy to determine the percentage of infiltrating T cells and MDSCs presented in Fig. 1C-K, 2C-F. (B) Gating strategy to determine the infiltrating CD8$^+$ T cells presented in Fig. 2H,K. (C) Gating strategy to determine the percentage and Ki67 expression of transferred T cells in Rag$^{-/-}$ hosts within tumor microenvironment presented in Fig. 4D-F, H. (D) Gating strategy to determine the percentage of IFN-γ-secreting transferred CD8$^+$ T cells in Rag$^{-/-}$ hosts within tumor microenvironment presented in Fig. 4G. (E) Gating strategy to determine the transferred CD45.2$^+$CD8$^+$ T cells in DLNs of CD45.1 hosts presented in Fig. 5J.

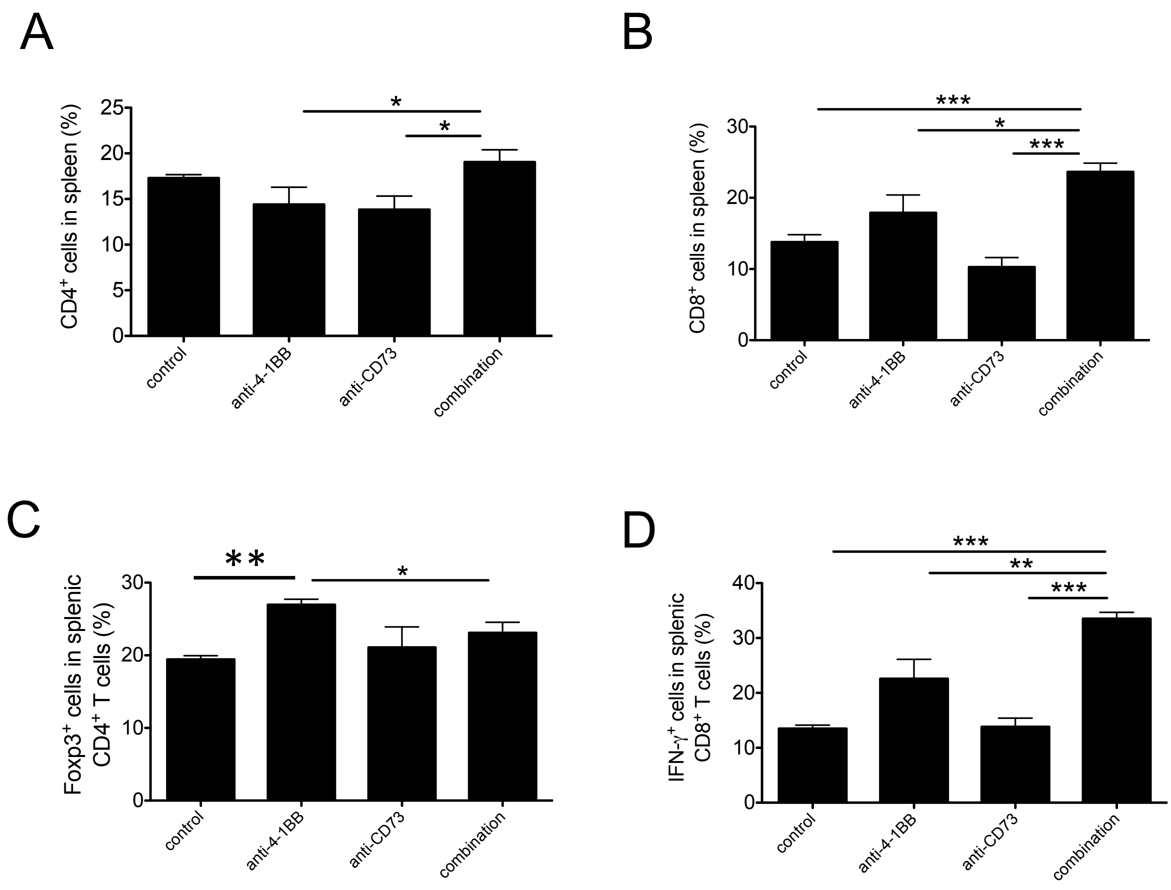

Supplementary Figure 2. Combination therapy of CD73 blockade and anti-4-1BB increases effector CD8$^+$ T cell accumulation in spleen of tumor-bearing mice. WT mice were injected s.c. with B16-SIY melanoma cells and treated with control IgG, anti-CD73, anti-4-1BB, or both anti-CD73 and anti-4-1BB. Percentage of CD4$^+$ (A) or CD8$^+$ (B) T cells in spleen of treated B16-SIY-bearing mice. Percentage of Foxp3$^+$ among CD4$^+$ T cells (C) or IFN-γ^+ among CD8$^+$ T cells (D) in spleen of treated B16-SIY-bearing mice. *; $p<0.05$, **; $p<0.01$. Data (mean ± SEM) are representative of at least 2 independent experiments with 3-5 independently analyzed mice/group.

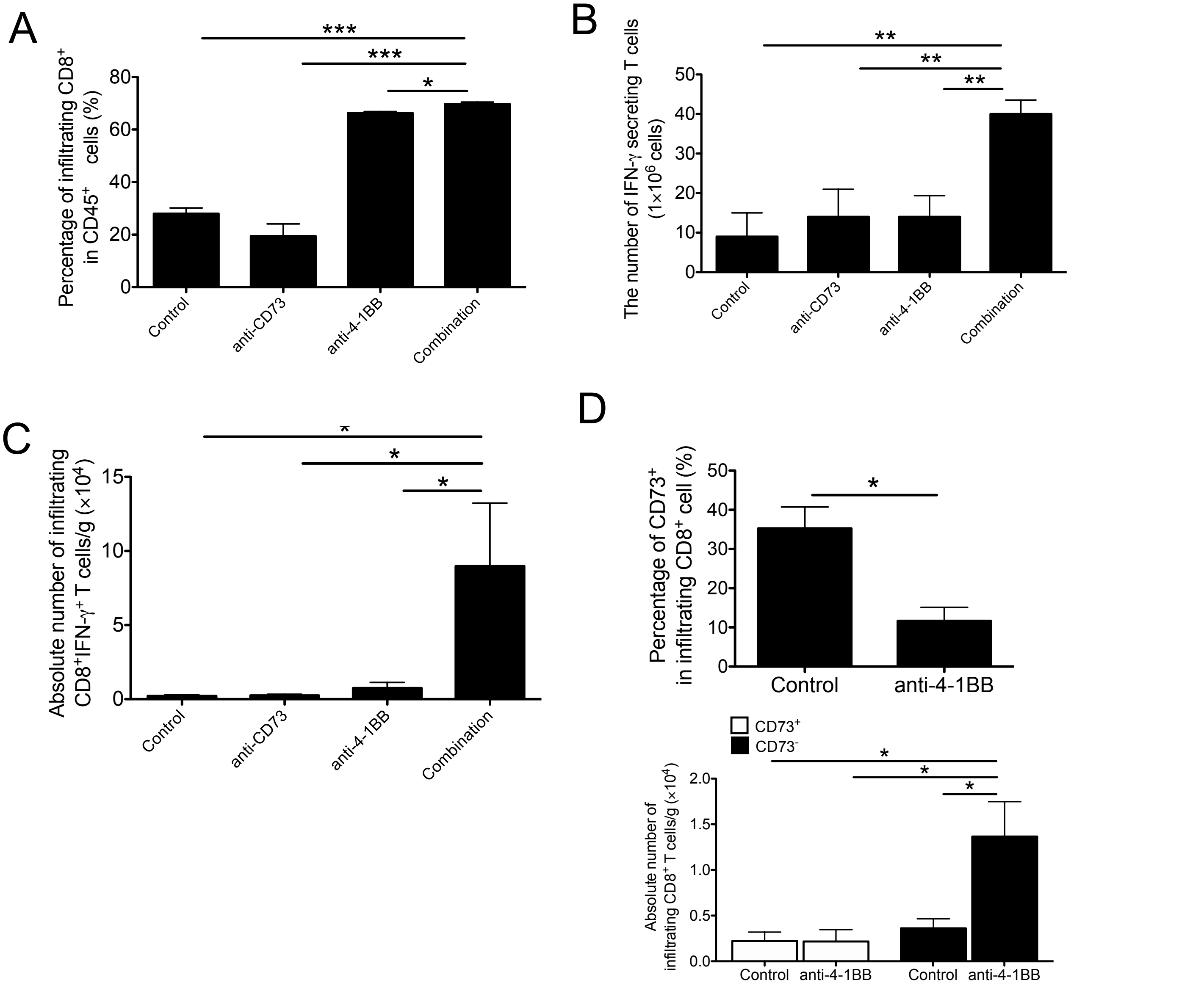

Supplementary Figure 3. Combination therapy of CD73 blockade and anti-4-1BB increases effector $CD8^+$ T cell activity in tumors at similar sizes. WT mice were injected s.c. with B16-SIY melanoma cells and treated with control IgG, anti-CD73, anti-4-1BB, or both anti-CD73 and anti-4-1BB. Mice were sacrificed 14 d after tumor inoculation with comparable tumor sizes. (A) Percentage of $CD8^+$ among $CD45^+$ tumor infiltrates in B16-SIY-bearing mice treated as indicated. (B) ELISPOT analysis for detection of IFN-γ–secreting $CD8^+$ cells from DLN of treated B16-SIY-bearing mice in the presence of SIY peptides (5 μg/ml). (C) Absolute number of $CD8^+IFN\text{-}\gamma^+$ T cells per gram of tumors were also calculated. (D) Percentage of $CD73^+$ among tumor-infiltrating $CD8^+$ T cells in B16-SIY-bearing mice treated as indicated. Absolute number of infiltrating $CD8^+CD73^+$ subset and $CD8^+CD73^-$ subset per gram of tumors were also calculated. *; p<0.05, **; p<0.01, ***; p<0.001. Data (mean ± SEM) are representative of 2 independent experiments with 3-5 independently analyzed mice/group.

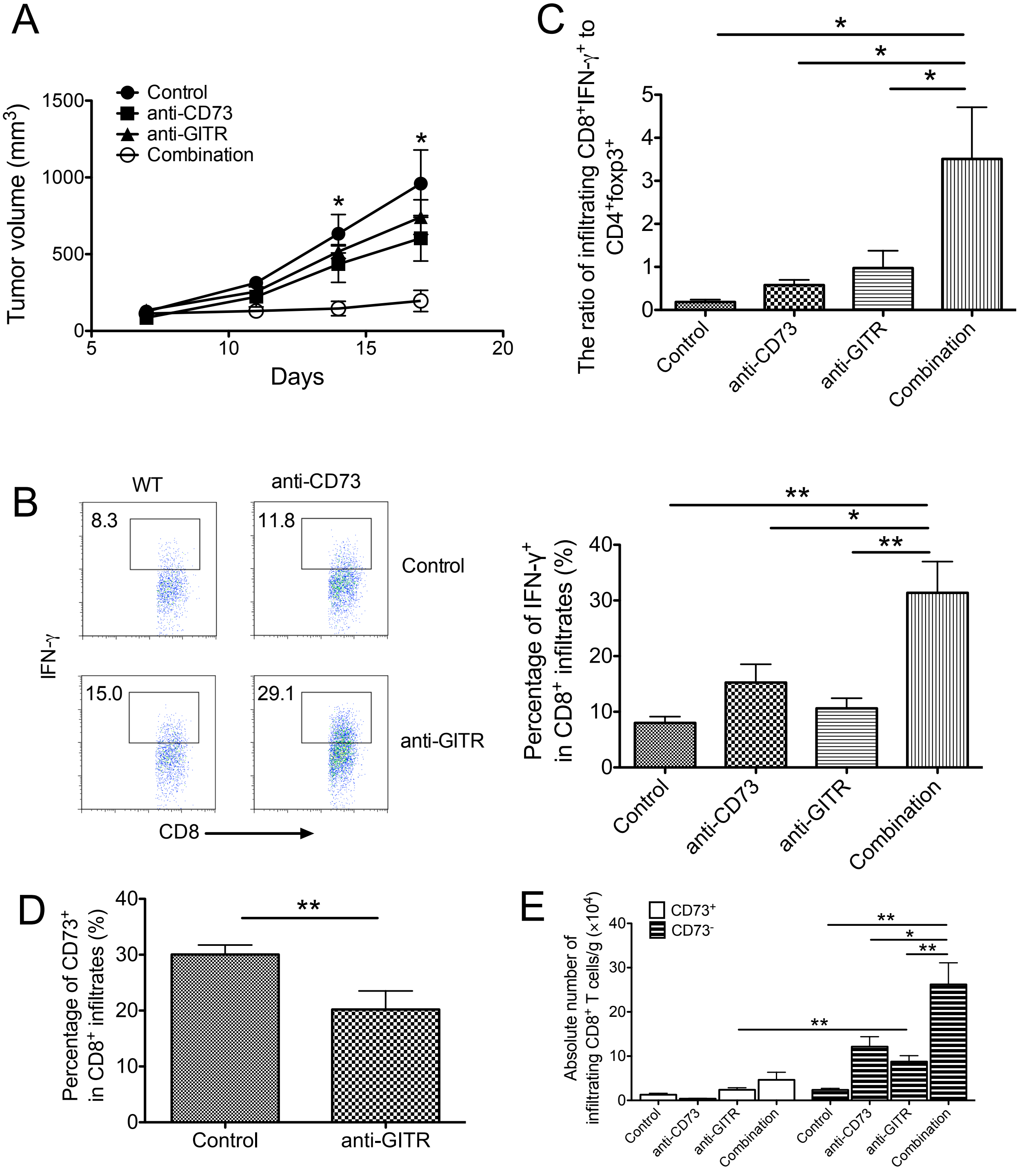

Supplementary Figure 4. Combination therapy of CD73 blockade and anti-GITR facilitates tumor regression with increased effector $CD8^+$ T cell activity. WT mice were injected s.c. with B16-SIY melanoma cells and treated with control IgG, anti-CD73, anti-GITR, or both anti-CD73 and anti-GITR. (A) Tumor size was measured every 3-4 d (5 mice per group). (B) Percentage of IFN-γ^+ or $CD8^+$ among tumor-infiltrating $CD3^+CD8^+$ T cells in the treated B16-SIY-bearing mice as indicated. (C) The ratio of $CD8^+$IFN-γ^+ to $CD4^+Foxp3^+$ Tregs was calculated. (D) The frequency of $CD73^+$ subset in $CD8^+$ tumor infiltrates from the treated B16-SIY-bearing mice as indicated. (E) Absolute number of $CD8^+CD73^+$ and $CD8^+CD73^-$ T cells per gram of tumors were calculated. *; $p<0.05$, **; $p<0.01$. Data (mean ± SEM) are representative of 2 independent experiments with 5 independently analyzed mice/group.

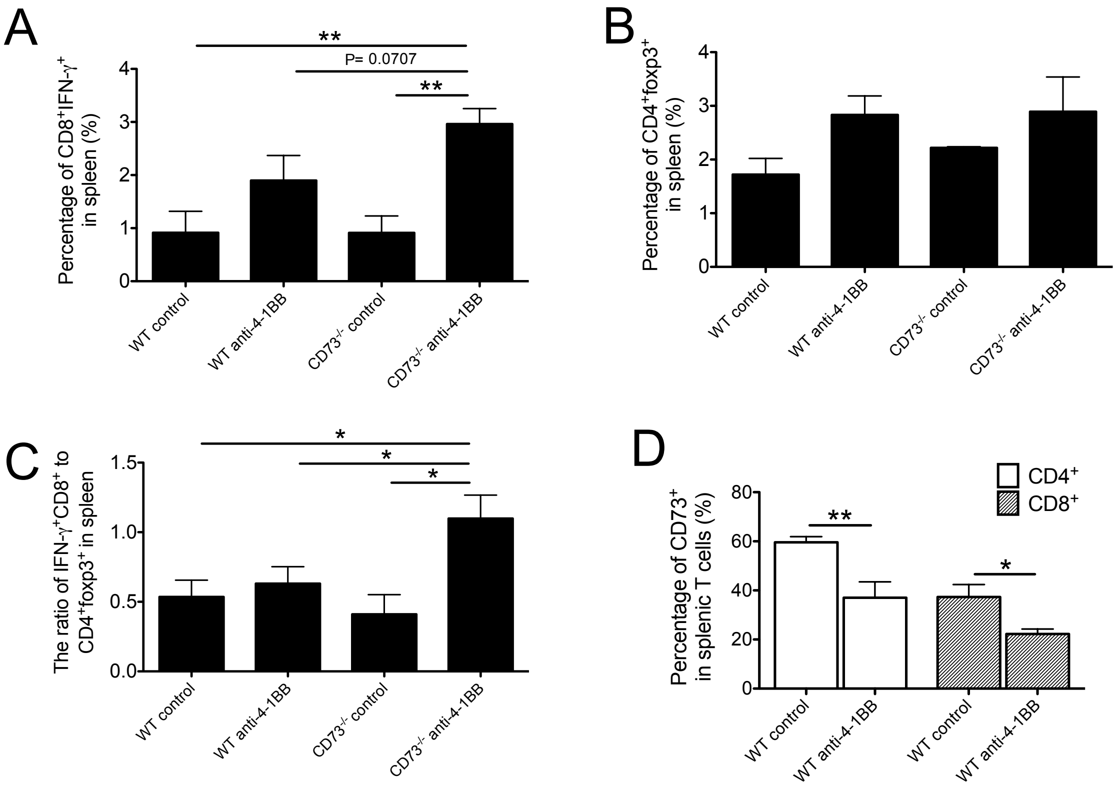

Supplementary Figure 5. B16-SIY tumor-bearing Rag1$^{-/-}$ mice receiving WT or CD73$^{-/-}$ pan-T cells were treated with control IgG or anti-4-1BB. Percentage of splenic CD8^{+}IFN-γ^{+} cells (A) and CD4^{+}Foxp3^{+} cells (B) in treated B16-SIY-bearing Rag1$^{-/-}$ mice were assessed by flow cytometry. (C) The ratio of CD8^{+}IFN-γ^{+} to CD4^{+}Foxp3^{+} Tregs was calculated. (D) The percentage of CD73^{+} in splenic CD4^{+} or CD8^{+} T cells was determined in treated B16-SIY-bearing Rag1$^{-/-}$ mice. *; $p<0.05$, **; $p<0.01$. Data (mean ± SEM) are representative of 2 independent experiments with 3-5 independently analyzed mice/group.

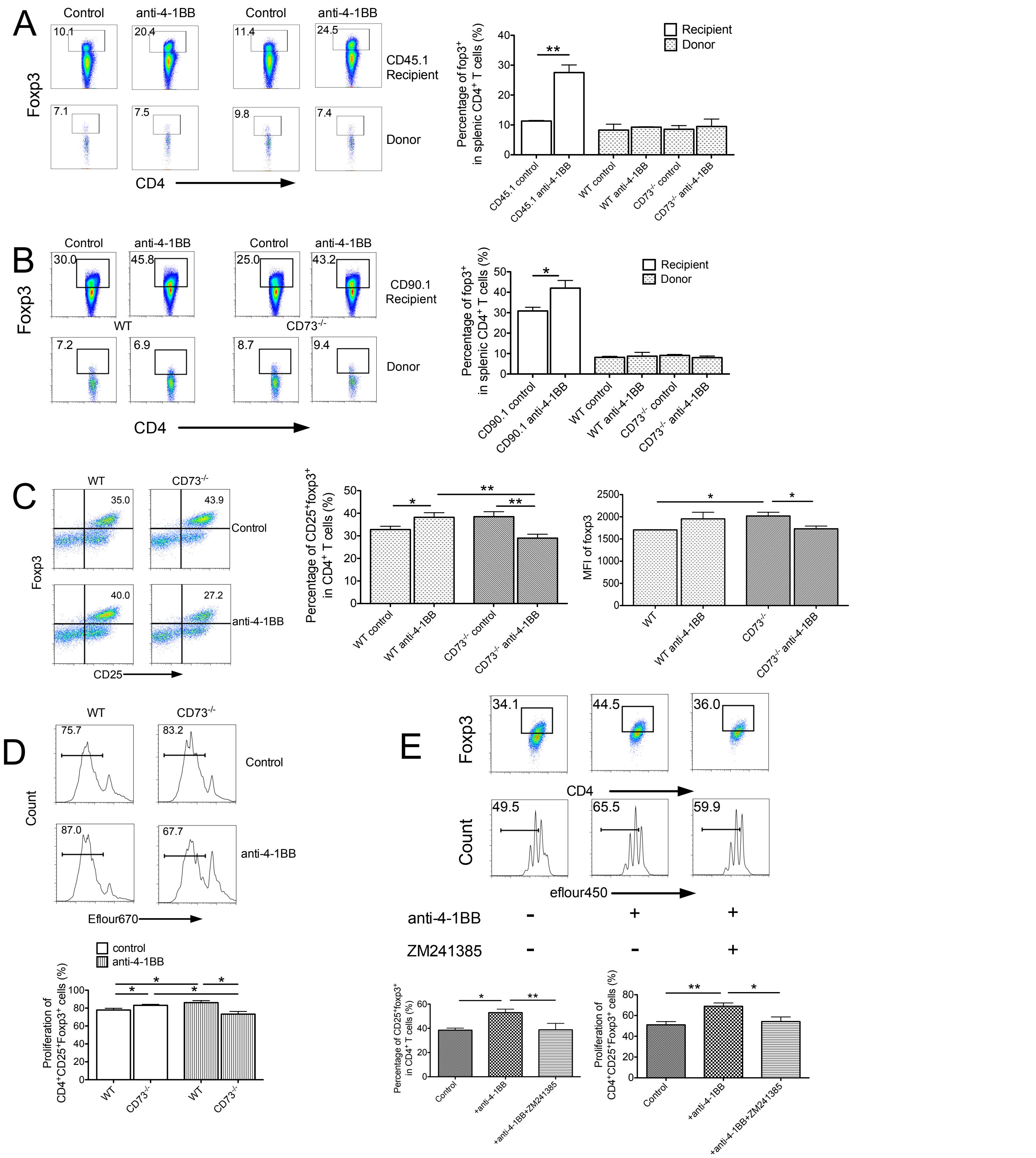

Supplementary Figure 6. CD73 deficiency impairs the expansion of Treg in the presence of anti-4-1BB. (A) $CD4^{+}CD25^{-}$ T cells from either $CD73^{+/+}$ or $CD73^{-/-}$ mice ($CD45.2^{+}$) were i.v. transferred into MC38-HS tumor bearing CD45.1 mice. Percentage of $Foxp3^{+}$ in recipient $CD4^{+}$ T cells ($CD45.1^{+}$) versus donor $CD4^{+}$ T cells ($CD45.2^{+}$) was analyzed 14 d after tumor challenge. (B) $CD4^{+}CD25^{-}$ T cells from either $CD73^{+/+}$ or $CD73^{-/-}$ OT-II mice ($CD90.2^{+}$) were i.v. transferred into B16-OVA tumor bearing CD90.1 mice. Percentage of $Foxp3^{+}$ in recipient $CD4^{+}$ T cells ($CD90.1^{+}$) versus donor $CD4^{+}$ T cells ($CD90.2^{+}$) was analyzed 14 d after tumor challenge. (C) WT or $CD73^{-/-}$ splenocytes labeled with proliferating dye eFlour450 were cultured with anti-CD3, TGF-β and IL-2 in the presence of plus control IgG or anti-4-1BB. After 5 d, the percentage of $CD25^{+}Foxp3^{+}$ Tregs among total $CD4^{+}$ T cells was analyzed by flow cytometry. (D) The proliferation of Treg population was evaluated by eFlour450 dilution using flow cytometry. (E) The WT splenocytes were treated as in (C), in the presence or absence of A2AR antagonists ZM241385. The percentage and eFlour450 dilution of $CD25^{+}Foxp3^{+}$ Tregs were subsequently analyzed by flow cytometry. *; $p<0.05$, **; $p<0.01$. Data (mean ± SEM) are representative of 2 independent experiments with 3-5 independently analyzed mice/group.

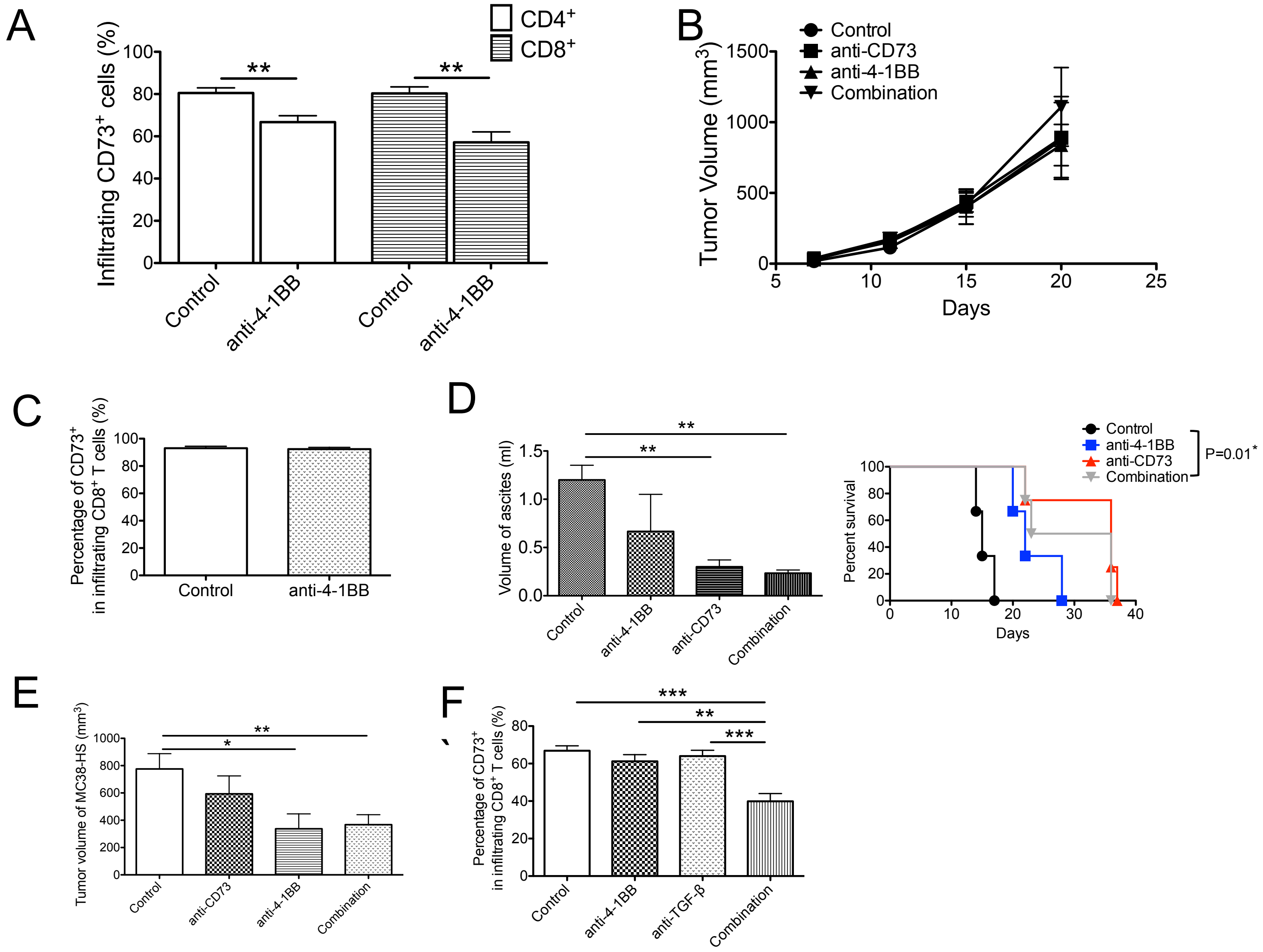

Supplementary Figure 7. TGF-β-rich tumor microenvironment dictates resistance to anti-4-1BB treatment in decreasing CD73⁺ subset in CD8⁺ T cell infiltrates. (A) LLC1-bearing bearing mice were treated with control IgG, or anti-4-1BB. The percentage of $CD73^+$ in tumor-infiltrating $CD8^+$ and $CD4^+$ T cells in treated tumor-bearing mice was analyzed by flow cytometry. **; $p<0.01$. Data (mean ± SEM) are representative of 2 independent experiments with 5 independently analyzed mice/group. (B) LLC1-bearing mice (n=5 per group) were treated with control IgG, anti-4-1BB, anti-CD73, or both anti-4-1BB/anti-CD73. Tumor growth was measured every 4-5 days. (C) The percentage of $CD73^+$ in tumor-infiltrating $CD8^+$ T cells from ID8-bearing mice treated with control IgG or anti-4-1BB was analyzed by flow cytometry. (D) Mice were injected i.p. with ID8 tumor cells and treated with control IgG, anti-4-1BB, anti-CD73, or both anti-4-1BB/anti-CD73. The ascites volume was measured 45 d after tumor challenge. The survival of each tumor-bearing mouse was recorded. (E) MC38-HS-bearing mice were treated with control IgG, anti-4-1BB, anti-CD73, or both anti-4-1BB/anti-CD73. The tumor size was measured 34 d after tumor challenge. (F) MC38-HS-bearing mice were treated with control IgG, anti-4-1BB, anti-TGF-β, or both anti-4-1BB/anti-TGF-β. The percentage of $CD73^+$ in tumor-infiltrating $CD8^+$ T cells in treated MC38-HS-tumor-bearing mice was analyzed by flow cytometry. *; $p<0.05$; **; $p<0.01$, ***; $p<0.001$. Data (mean ± SEM) are representative of 2 independent experiments with 5 independently analyzed mice/group.

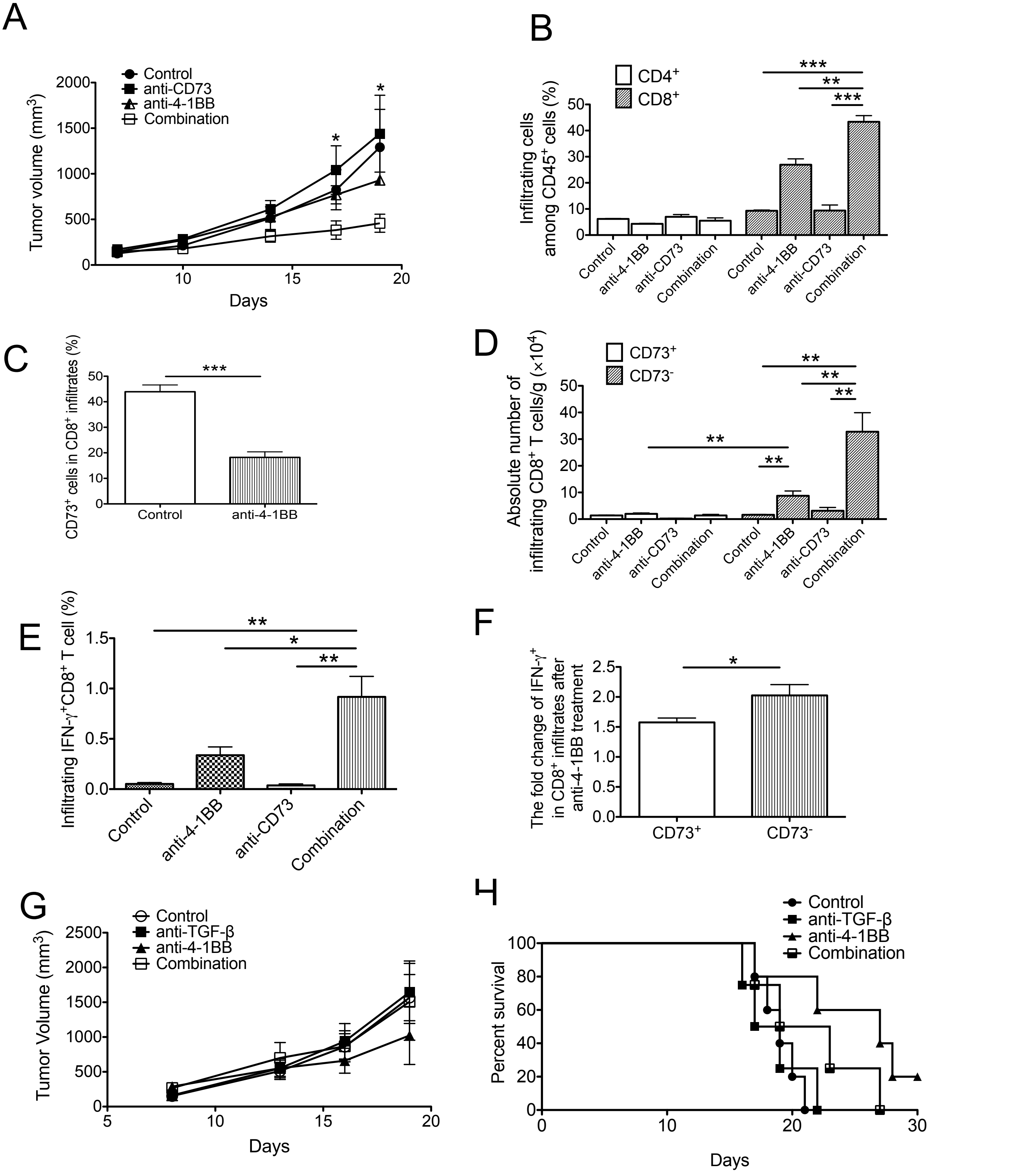

Supplementary Figure 8. Combination therapy of CD73 blockade and anti-4-1BB facilitates TGF-β-low tumor regression with increased effector CD8+ T cell activity. WT mice were injected s.c. with MC38-AS cells and treated with anti-4-1BB, anti-CD73, anti-4-1BB/anti-CD73 or control IgG. (A) Tumor size was measured every 2-4 d. (B) MC38-AS tumors from treated WT mice were harvested 19 d after tumor challenge and analyzed by flow cytometry for accumulation of $CD3^+CD4^+$ and $CD3^+CD8^+$ T cells. (C) Percentage of $CD73^+$ cells among tumor-infiltrating $CD8^+$ T cells. (D) Absolute number of $CD8^+CD73^+$ and $CD8^+CD73^-$ T cells per gram of tumors were calculated. (E) Percentage of IFN-γ^+CD8^+ T cells within tumor microenvironment. (F) Anti-4-1BB therapy resulted in an increase in the number of $CD73^-$ subset preferentially producing IFN-γ. *; p<0.05, **; p<0.01, ***; p<0.001. Mice were injected with B16-SIY tumor cells and treated with control IgG, anti-4-1BB, anti-TGF-β, or both anti-4-1BB/anti-TGF-β. (G) Tumor size was measured every 3 d, and (H) mice survival was recorded till day 30.

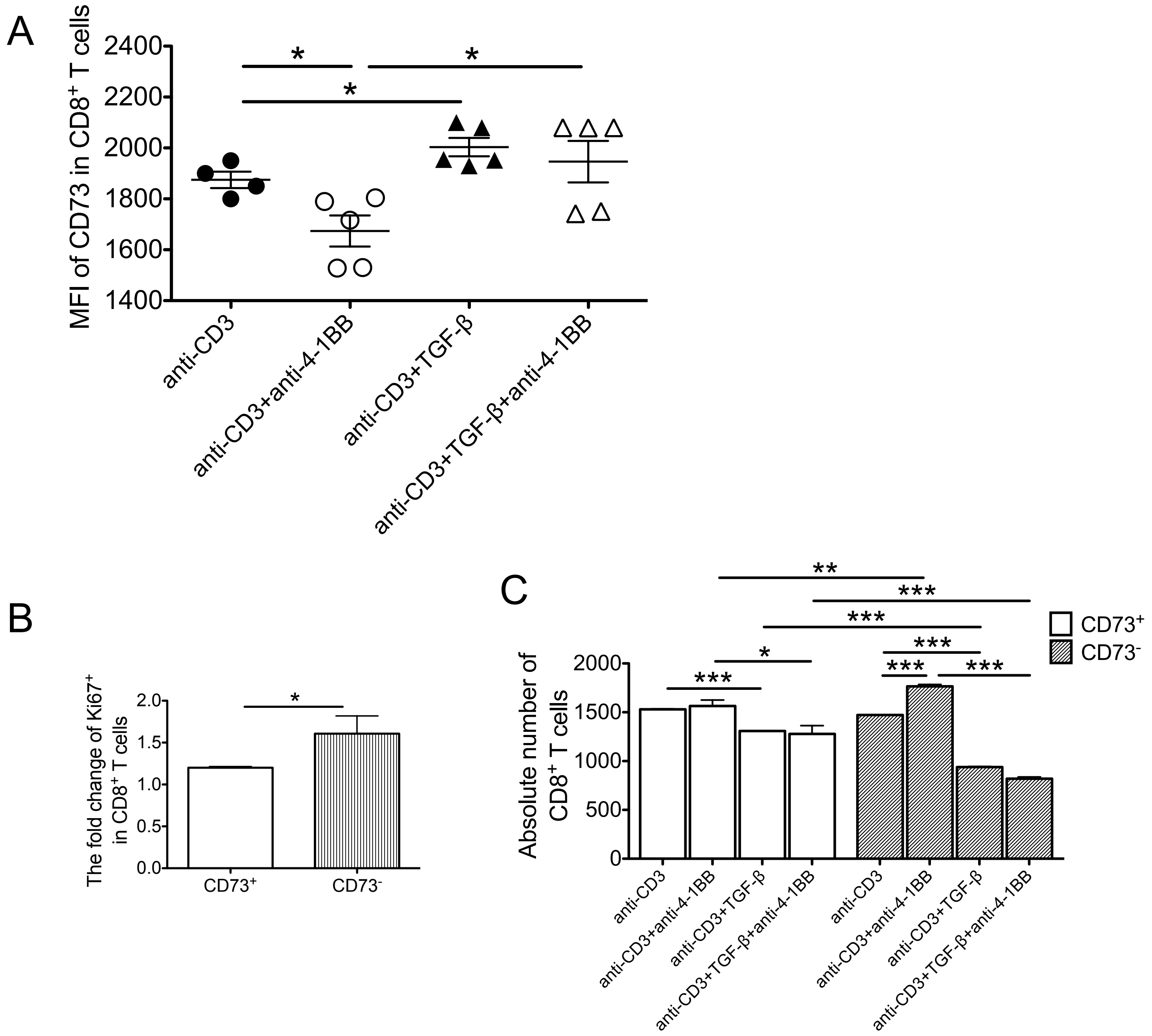

Supplementary Figure 9. TGF-β sustains CD73 expression and inhibits the expansion of human CD8$^+$ T cells in response to 4-1BB-mediated costimulation. (A) Human PBMCs were cultured with TGF-β, anti-4-1BB, TGF-β/anti-4-1BB or control IgG in the presence of anti-CD3. After 3 d, the expression levels of CD73 (A) and Ki67 (B) in CD8$^+$ T cells were measured by flow cytometer. (C) The absolute number of CD73$^+$ versus CD73$^-$ subsets among these treated CD8$^+$ T cells were counted. *; p<0.05, **; p<0.01, ***; p<0.001. Data (mean ± SEM) are representative of 2 independent experiments.

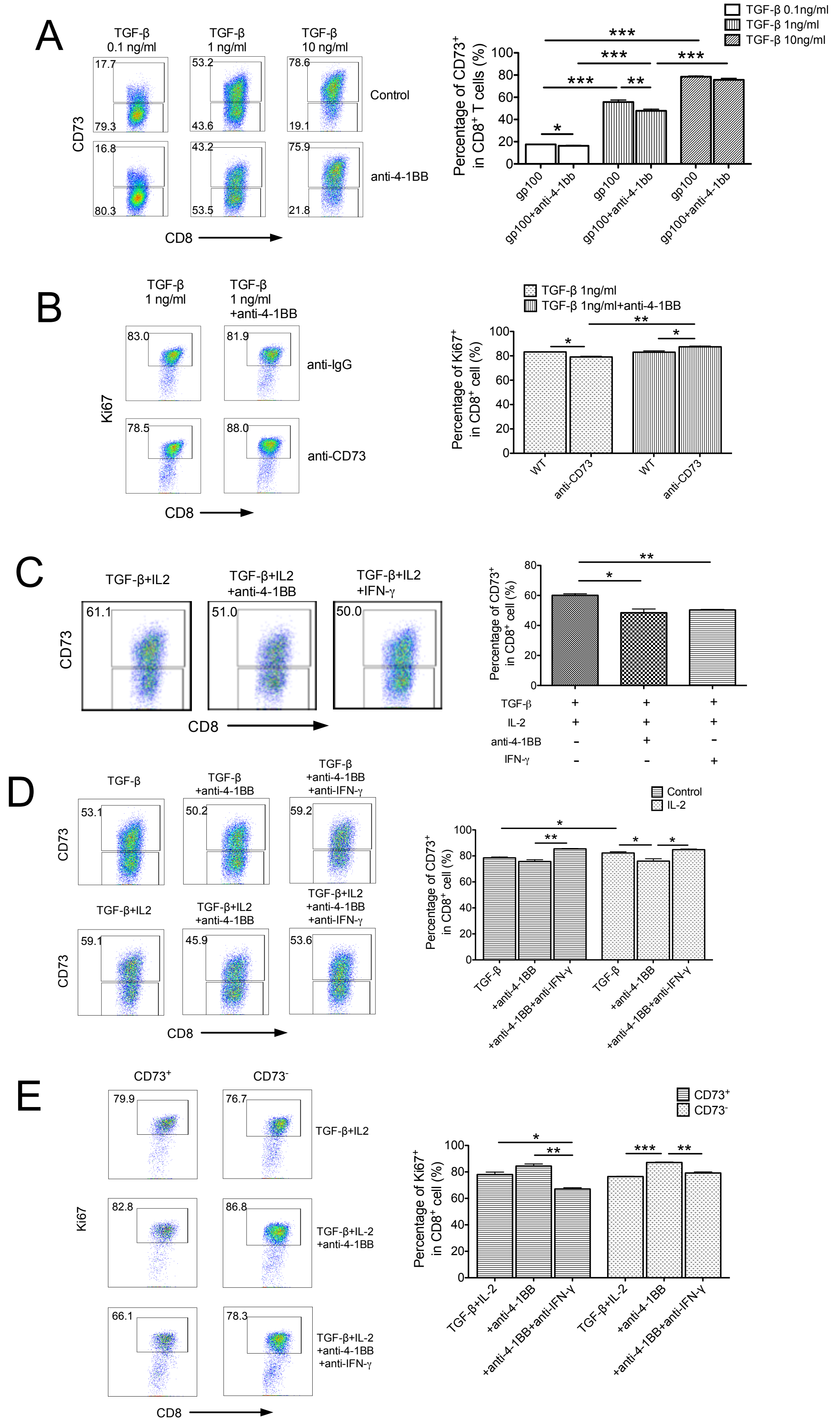

Supplementary Figure 10. Purified CD8+ T cells from Pmel mice were cultured with different doses of TGF-β, anti-4-1BB, TGF-b/anti-4-1BB or control IgG in the presence of gp100/anti-CD28. After 3 d, the percentage of CD73+ (A) or Ki67+ (B) in CD8+ T cells was measured by flow cytometer. Purified CD8+ T cells were cultured with TGF-β, anti-4-1BB, IL-2, IFN-γ anti-IFN-γ or control IgG in the presence of gp100/anti-CD28. After 3 d, percentage of CD73+ (C, D) or Ki67+ (E) in CD8+CD73+ versus CD8+CD73- subsets under indicated conditions was compared by flow cytometry. *; p<0.05, **; p<0.01, ***; p<0.001. Data (mean ± SEM) are representative of 2 independent experiments.

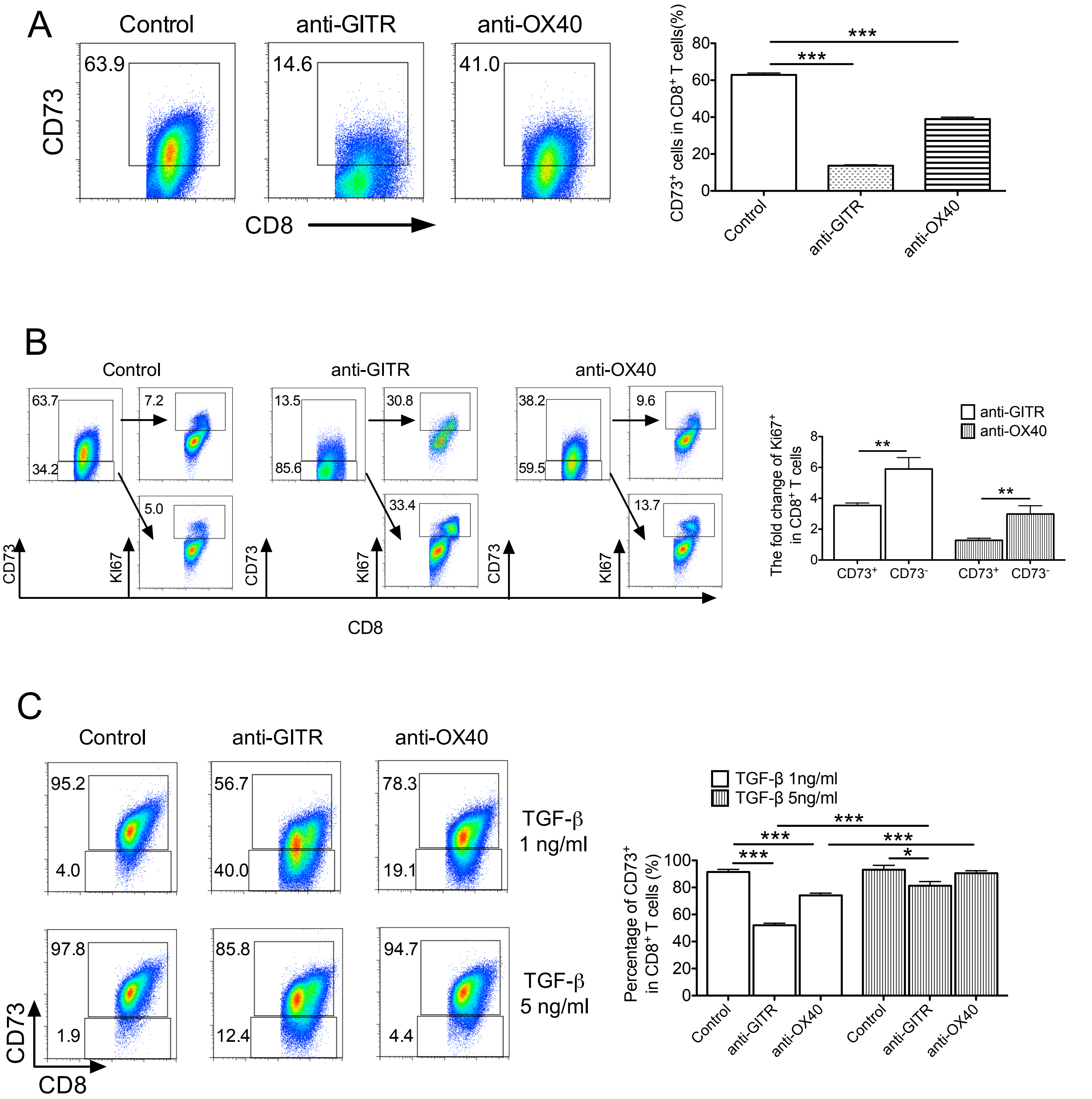

Supplementary Figure 11. The inhibitory effect of TGF-β on GITR- or OX40-mediated regulation of CD73 expression and expansion of CD8⁺ T cells. (A) Purified CD8⁺ T cells were cultured with agonistic anti-GITR, anti-OX40 or control IgG in the presence of anti-CD3/anti-CD28. After 3 d, the percentage of CD73⁺ in CD8⁺ T cells was measured by flow cytometer. (B) The percentages of Ki67⁺ in CD73⁺ versus CD73⁻CD8⁺ T cell subsets were compared. (C) Purified CD8⁺ T cells were cultured with TGF-β, anti-GITR, TGF-β/anti-GITR, anti-OX40, TGF-β/anti-OX40 or control IgG in the presence of anti-CD3/anti-CD28. After 3 d, the percentage of CD73⁺ in CD8⁺ T cells was measured by flow cytometer. *; p<0.05, **; p<0.01, ***; p<0.001.

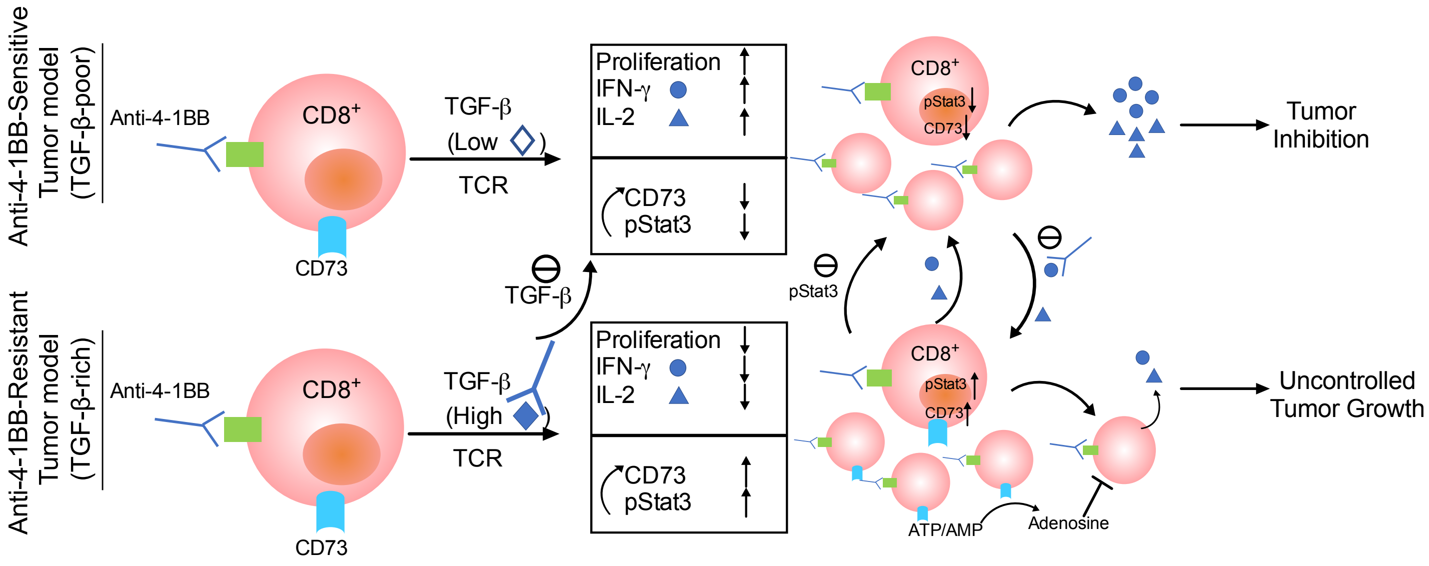

Supplementary Figure 12. Mechanism of action and resistance of agonistic anti-4-1BB cancer immunotherapy.
